# Genome-Wide Identification of *Triticum aestivum* Xylanase Inhibitor Gene Family and Inhibitory Effects of *XI-2* Subfamily Proteins on *Fusarium graminearum* GH11 Xylanase

**DOI:** 10.3389/fpls.2021.665501

**Published:** 2021-07-26

**Authors:** Yang Liu, Nannan Han, Sheng Wang, Can Chen, Jie Lu, Muhammad Waheed Riaz, Hongqi Si, Genlou Sun, Chuanxi Ma

**Affiliations:** ^1^College of Agronomy, Anhui Agricultural University, Hefei, China; ^2^Key Laboratory of Wheat Biology and Genetic Improvement on Southern Yellow and Huai River Valley, Ministry of Agriculture and Rural Affairs, Hefei, China; ^3^Biology Department, Saint Mary’s University, Halifax, NS, Canada; ^4^National United Engineering Laboratory for Crop Stress Resistance Breeding, Hefei, China; ^5^Anhui Key Laboratory of Crop Biology, Hefei, China

**Keywords:** *TaXI* gene family, xylanase inhibitor, expression profiles, enzyme activity, inhibition of cell necrosis

## Abstract

*Triticum aestivum* xylanase inhibitor (*TaXI*) gene plays an important role in plant defense. Recently, *TaXI–III* inhibitor has been shown to play a dual role in wheat resistance to *Fusarium graminearum* infection. Thus, identifying the members of the *TaXI* gene family and clarifying its role in wheat resistance to stresses are essential for wheat resistance breeding. However, to date, no comprehensive research on *TaXIs* in wheat (*Triticum aestivum L.*) has been conducted. In this study, a total of 277 *TaXI* genes, including six genes that we cloned, were identified from the recently released wheat genome database (IWGSC RefSeq v1.1), which were unevenly located on 21 chromosomes of wheat. Phylogenetic analysis divided these genes into six subfamilies, all the six genes we cloned belonged to *XI-2* subfamily. The exon/intron structure of most *TaXI* genes and the conserved motifs of proteins in the same subfamily are similar. The *TaXI* gene family contains 92 homologous gene pairs or clusters, 63 and 193 genes were identified as tandem replication and segmentally duplicated genes, respectively. Analysis of the *cis-*acting elements in the promoter of *TaXI* genes showed that they are involved in wheat growth, hormone-mediated signal transduction, and response to biotic and abiotic stresses. RNA-seq data analysis revealed that *TaXI* genes exhibited expression preference or specificity in different organs and developmental stages, as well as in diverse stress responses, which can be regulated or induced by a variety of plant hormones and stresses. In addition, the qRT-PCR data and heterologous expression analysis of six *TaXI* genes revealed that the genes of *XI-2* subfamily have double inhibitory effect on GH11 xylanase of *F. graminearum*, suggesting their potential important roles in wheat resistance to *F. graminearum* infection. The outcomes of this study not only enhance our understanding of the *TaXI* gene family in wheat, but also help us to screen more candidate genes for further exploring resistance mechanism in wheat.

## Introduction

The plant cell wall is a complex network structure composed of cellulose, hemicellulose, lignin, pectin, and protein, which is the main physical barrier against pathogen attack (Walton, 1994; Have et al., 2002). In order to absorb plant tissue nutrients and successfully colonize host tissue, pathogens secrete a variety of cell wall degrading enzymes (CWDEs) to breach the cell wall barrier of plants during the infection process (Cantu et al., 2008). In particular, endo-β-1,4-xylanase (EC3.2.1.8, also known as xylanase) is key enzyme in plant or microbial for the degradation of xylan (Wong et al., 1998; Collins et al., 2005), an important cell wall polysaccharide in cereals (Vogel, 2008). According to the sequence-based glycoside hydrolase (GH) classification (CAZy classification), most xylanases belong to the GH10 and GH11 families (Henrissat, 1991). The xylanase secreted by pathogens cannot only degrade xylan, but also induce plant tissue necrosis and defense response, which is independent of enzyme activity (Sharon et al., 1993; Enkerli et al., 1999; Furmanmatarasso et al., 1999; Belien et al., 2005; Noda et al., 2010). However, the activity of microbial xylanase can be inhibited by specific proteins (XIs, xylanase inhibitors) located in the plant cell wall (Dornez et al., 2010b).

In wheat, three structurally different classes of xylanase inhibitors (XIs) have been found: *Triticum aestivum* XI (TAXI) (Debyser et al., 1999), xylanase inhibitor (XIP), (Mclauchlan et al., 1999) and thaumatin-like XI (TLXI) (Fierens et al., 2007). The inhibition spectra of different kinds of inhibitors are different. The results showed that TAXI-type inhibitors are specific to xylanase of the GH11 family, but can’t inhibit the xylanase of the GH10 family (Gebruers et al., 2001). To date, six different TAXI-type XI gene sequences have been identified in common wheat, including *TaXI–IA*, *TaXI–IB*, *TaXI–IIA*, *TaXI–IIB*, *TaXI–III*, and *TaXI–IV* (Fierens et al., 2003; Igawa et al., 2004; Raedschelders et al., 2005). The coding region sequences of *TaXI–IB* and *TaXI–IIB* show highly homology to those of *TaXI–III* (99.6% homology) and *TaXI–IV* (99.8% homology), respectively. Thus, TAXI–III and TAXI–IV are considered as the isomer of TAXI–IB and TAXI–IIB, respectively (Dornez et al., 2010b).

At present, all known TAXI-type inhibitors have similar characteristics with an alkaline isoelectric point PI > 8.0, and exist in two molecular forms: a monomer form with a molecular mass of approximately 40 kDa; and a processed form consisting of two small peptides of approximately 10 and 30 kDa, which are held together by one or more intermolecular disulfide bond (Dornez et al., 2010b). The X-ray crystal structure showed that the TAXI-type inhibitors consist of a two β-barrel domains divided by an extended open cleft (Sansen et al., 2004a). And TAXI-type inhibitors display structural homology with pepsin-like aspartic peptidases with low sequence similarities, and lack of proteolytic activity (Sansen et al., 2004b).

Xylanase inhibitors can specifically inhibit microbial xylanases, and can be induced by pathogens infection, so they are considered to be part of the plant defense mechanism against microbial pathogens. Previous study found that TAXI–I inhibitor has inhibitory effects on GH11 family xylanase of *Bacillus subtilis* (BSX) and *Aspergillus niger* (ANX), TAXI–II inhibitor can inhibit the activity of BSX (Gebruers et al., 2001). The activity of two xylanases [XylA (FGSG_10999) and XylB (FGSG_03624)] of *F. graminearum* heterologously expressed in *Escherichia coli* or *Pichia pastoris* can be inhibited by TAXI–I and TAXI–III inhibitors (Belien et al., 2005; Moschetti et al., 2013). When plants are infected or injured by pathogens, genes involved in defense will be induced to express. After wheat spikes were infected by *F. graminearum*, or wheat leaves were infected by powdery mildew, the expression of *TaXI–I* and *TaXI–III/IV* were up-regulated (Igawa et al., 2004). Analogously, the contents of TAXI–IA (40 KDa) and TAXI–IIA (40 KDa) proteins in wheat were increased by 2–3 times after 5 days of inoculation with the *F. graminearum* mutant △Tri5, and the content of TAXI–IA protein was increased by five times after 15 days of infection (Dornez et al., 2010a). Recent studies have also found that TAXI–III inhibitor can inhibit not only the degradation of cell wall xylan by *F. graminearum* xylanase FGSG_03624 and FGSG_10999, but also cell death caused by these xylanases (Moschetti et al., 2013; Moscetti et al., 2015; Tundoa et al., 2015). Moreover, transgenic durum wheat with *TaXI–III* gene can delay the symptoms of wheat *Fusarium* head blight (FHB) (Moschetti et al., 2013).

Although, the functions of *TaXI–III* gene have been described in more detail in wheat (Igawa et al., 2004; Moschetti et al., 2013; Moscetti et al., 2015; Tundoa et al., 2015), the functions of other *TaXI*-type genes are not fully understood. Therefore, the whole genome analysis of *TaXI* genes is essential to further understand the function of the wheat *TaXI* gene family. In the present study, we identified 277 *TaXI* genes from the newly published wheat reference genome database through genome-wide search. These predicted genes were systematically analyzed, including subcellular location, chromosome location, phylogenetic relationship, gene duplication, gene structure, protein conserved motifs, *cis-*acting elements, and expression profiles, to explore the evolutionary relationship and functional characteristics of these genes. To better understand the role of *TaXI* genes in wheat resistance to *F. graminearum* infection, we used qRT-PCR method to determine the expression levels of six *TaXI* genes. The inhibitory effect of these XIs on *F. graminearum* xylanase was characterized by heterologous expression in *P. pastoris.* These studies not only help us to further understand the evolution and functions of *TaXI*-type genes, but also screen more candidate genes. The results will lay the foundation for characterizing the resistance mechanism to *F. graminearum* in wheat.

## Materials and Methods

### Genome-Wide Identification of *TaXI* Genes in Wheat

The latest reference genome of wheat and HMM profiles of TAXI (PF14543 and PF14541) were downloaded from the wheat genome database IWGSC (IWGSCRefSeqannotionv1.1^1^) (Appels et al., 2018) and PFAM database^2^ (Elgebali et al., 2019). The hmmsearch program in the HMMER software (Jaina et al., 2013) was used to search the TAXI HMM profile in the wheat genome, and the reliable results were screened out based on E-value less than or equal to 1 × 10^–10^ for constructing the wheat-specific TAXI HMM profile by hmmbuild program, and then searching the wheat reference genome again with an E-value threshold 1 × 10^–10^. To avoid missing other members of the *TaXI* gene family, the TAXI- type protein sequences from wheat, durum wheat, rye and barley reported on NCBI website were used as query sequences for BLASTP alignment with the E-value threshold of 1 × 10^–5^. Then, we combined the predicted genes from the two methods and removed the duplications. Only the most reliable one was retained from multiple transcripts of the *TaXI* gene. The protein sequences of the putative genes were submitted to NCBI–CDD^3^ and PFAM online databases for verification, and the putative genes without the TAXI_N and TAXI_C domains were excluded. These presumed genes with the E value greater than or equal to 1 × 10^–20^ predicted by Pfam database were deleted. Finally, the member of the wheat *TaXI* gene family with high confidence was obtained. All the final presumed TAXI protein sequences were submitted to Expasy online software^4^ (Artimo et al., 2012) to calculate the number of amino acids, molecular weight and theoretical isoelectric point. The signal peptide structure of TAXI proteins was predicted by using SignalP 4.0 online website^5^. The subcellular localization of each member of the *TaXI* gene family was predicted by using CELLOv2.5 online software^6^ (Yu et al., 2006).

### Analysis of Chromosome Location and *Cis-*acting Regulatory Elements

The physical location of each presumed *TaXI* genes on the chromosomes was searched from the wheat genome database published in IWGSC. The physical map of wheat *TaXI* genes on the chromosomes of common wheat was drawn by using TBtools (Chen et al., 2020). The 2.0 kb upstream genomic sequences of all *TaXI* transcripts were extracted from the reference genome of wheat, and these sequences were submitted to the PlantCare database^7^ to predict the *cis-*acting element on the promoter. The distribution map of *cis-*acting elements was drawn by using TBtools (Chen et al., 2020).

### Multiple Sequence Alignment and Phylogenetic Analysis

Multiple sequence alignment of the amino acid sequences of TAXI proteins, including the reported TAXI-type protein sequences of wheat, durum wheat, rye and barley from NCBI website^8^ and the speculated wheat TAXI protein sequences in this study, were performed using ClustalW tool^9^ (Larkin et al., 2007). The phylogenetic tree was constructed by MEGA7.0 software using the neighbor-joining method (NJ) (Kumar et al., 2016). The bootstrap tests were conducted with 1,000 replicates and other parameters were set to default.

### Analysis of Gene Duplication

Tandem duplication means that multiple members of a gene family appear in the same or adjacent spacer region (Ramamoorthy et al., 2008). In this study, the tandem duplication of *TaXI* genes in wheat was analyzed according to the following three criteria: (a) the alignment covered >70% of the longer gene; (b) the aligned region had an identity >70% (Gu et al., 2002; Yang S. et al., 2008); and (c) the adjacent homologous genes are on the same chromosome, in which no more than one gene is inserted (Zhu et al., 2014). The BLASTN program and the method described by Wang et al. (2016) were used to determine the tandem duplication of the *TaXI* genes in wheat. The segmental duplication of genes was produced by polyploidy and chromosome rearrangement (Yu et al., 2005). McscanX software (Wang et al., 2012) was used to determine the segmental duplication of wheat *TaXI* genes, and the TBtools (Chen et al., 2020) was used to display the results of segmental duplication. To explore the mechanism of gene differentiation after duplication, multiple sequences were aligned using MEGA7.0. Then the ratio of non-synonymous nucleotide substitution to synonymous nucleotide substitution (ka/ks) between duplicated gene pairs was calculated by TBtools (Chen et al., 2020). The occurrence date of gene pairs duplication events was calculated by the equation *T* = Ks/2 λ × 10^–6^ Mya (λ = 6.5 × 10^–9^) (Yang Z. et al., 2008; Wang et al., 2017).

### Analysis of Gene Structure and Conserved Motif

According to the gene ID of wheat *TaXI* genes, the intron, exon and chromosome location of wheat *TaXI* genes were extracted from Ensembl plants^10^. To analyze other conserved motifs in the full-length amino acid sequence of TAXIs, the protein motifs were searched using the MEME online website^11^ (Timothy et al., 2006). The gene structure and protein motifs of the wheat *TaXI* gene family were visualized by TBtools (Chen et al., 2020).

### RNA-Seq Data Analysis

To analyze the expression profiles of wheat *TaXI* genes in different tissues and stress challenges, the RNA-seq data of the *TaXI* genes were downloaded from WheatExp^12^ and visualized with TBtools (Chen et al., 2020).

### Cloning of *TaXIs* and *FGSGs* Genes

The gene sequences of *TaXI* in the NCBI website, including *TaXI–IA* (AJ438880), *TaXI–IB* (AJ697851), *TaXI–IIA* (AJ697849), *TaXI–IIB* (AJ697850), *TaXI–III* (AB114627), and *TaXI-IV* (AB114628), and the corresponding *TaXI* gene sequences in the wheat reference genome were used to design specific primers to amplify these *TaXI* genes. Similarly, specific primers of *FGSGs* were designed according to the *FGSG_03624* (XM_011323775.1), *FGSG_10999* (XM_011327069.1), *FGSG_11304* (XM_011327638.1), and *FGSG_11487* (XM_011327424.1) gene sequences published in the NCBI website.

The genomic DNA and the cDNA of wheat spikes infected with *F. graminearum* of three whet cultivars, including Wangshuibai (WSB), Annong 1589 (AN1589), and Annong 8455 (AN8455), were used as the template for PCR amplification. The PCR amplification was performed using Phanta MaxSuper-Fidelity DNA Polymerase (Vazyme, China), according to the manufacturer’s manual. The PCR products were purified using FastPure^TM^ Gel DNA Extraction Mini Kit (Vazyme, China). The purified product was ligated with pBLUNT vector using 5 min ^TM^ TA/Blunt-Zero Cloning Kit (Vazyme, China), and then transformed into *E. coli* competent cell Trans1-T1 (Transgen Biotech, China). The positive clones were selected and sent to Shanghai Shenggong Biological Company for sequencing. The sequences were compared by DNAman tool, and the bacterial solution with correct sequence was preserved.

### Analysis of *TaXI* Genes Expression After Infection by *F. graminearum*

Common wheat WSB, AN1589, and AN8455 were planted in the field. Each wheat variety was injected with 15 μl of *F. graminearum* (mixed strain) conidia suspension or water between the lemma and palea of the wheat spikelet at anthesis. Samples were taken at 12, 24, 36, 48, and 72 h after inoculation, and frozen in liquid nitrogen and stored in the refrigerator at −80°C.

Total RNA was extracted from wheat spikelets infected by *F. graminearum* using RNAprep Pure Plant Plus Kit (Polysaccharides and Polyphenolics-rich) (TIANGEN, China) according to the manufacturer’s instruction. The total RNA was reverse transcribed into cDNA using HiScript^®^ IIQRT SuperMix for qPCR (+gDNAwiper) (Vazyme, China). The oligonucleotide primer pairs used in the qRT-PCR analyses were listed in the Supplementary Table 12. *Actin* (AB181991) was used as the internal reference gene. The qRT-PCR was carried out using AceQ^®^ Universal SYBR^®^ qPCRMasterMix (Vazyme, China) according to the manufacturer’s instructions. Each treatment was repeated three times. The relative expression was determined by using the 2^–Δ^ ^Δ^ ^*CT*^ method (Schmittgen and Livak, 2008).

### Heterologous Expression of *TaXIs* and *FGSGs* in *P. pastoris*

EasyPur^®^ Plasmid Mini-Prep Kit (TransgenBiotech, China) was used to extract plasmids from *E. coli* bacteria according to the manufacturer’s instruction. The coding regions without signal peptides were amplified from the plasmids, including pBlunt-*TaXI–IA*, pBlunt-*TaXI–IAb*, pBlunt-*TaXI–IIA*, pBlunt-*TaXI–IIAb*, pBlunt-*TaXI–III*, pBlunt-*TaXI–IV*, pBlunt-*FGSG_03624*, pBlunt-*FGSG_10999*, pBlunt-*FGSG_11304*, and pBlunt-*FGSG_11487*, by using the homologous recombination primer pairs (Supplementary Table 12). The PCR products were purified and ligated into the *Eco*RI and *Eag*I sites of the pPIC9K plasmid using Clon Express^®^ IIOneStep CloningKit (Vazyme, China) to obtain the recombinant expression vector pPIC9K-*TaXI–IA*, pPIC9K-*TaXI–IAb*, pPIC9K-*TaXI–IIA*, pPIC9K-*TaXI–IIAb*, pPIC9K-*TaXI–III*, pPIC9K-*TaXI–IV*, pPIC9K-*FGSG_03624*, pPIC9K-*FGSG_10999*, pPIC9K-*FGSG_11304*, and pPIC9K-*FGSG_11487*. Then these recombinant plasmids were linearized by restriction endonuclease *Sac*I/*Bsp*EI/*Sal*I (NEWENGLANDBioLabs, United States), respectively. The linearized plasmids were purified using TaKaRa Mini BEST DNA Fragment Purification Kit Ver.4.0 (TaKaRa, Japan).

According to the manufacturer’s instruction, *P. pastoris* GS115 competent cells were prepared by *P. pastoris* transformation kit (Coolaber, China), and the linearized plasmid was transformed into GS115 competent cells. Some positive colonies were tested by PCR with pPIC9KF1/pPIC9KR1 primers using 2x Rapid TaqMaster Mix (Vazyme, China). The correct colonies were cultured and induced with methanol to produce the secretory proteins as reported by Sella et al. (2013). After 1 week, liquid cultures were centrifuged at 10,000×*g* for 10 min. The supernatant was diluted with water (OD550 = 0.6–0.8) and used as the crude enzyme for determining enzyme activity.

### Detection of *TAXIs* Enzyme Activity

The xylanase inhibition activity of the crude enzyme was measured by radial gel diffusion assay or the DNS assay. Radial gel diffusion assay was performed according to the method reported by Emami and Hack (2001) with minor modification. Two portions of 100 μL xylanase were set up, one was added with 600 μL xylanase inhibitor, and another was added with 600 μL boiling inactivated xylanase inhibitor (100°C, 40 min). These mixtures were reacted at 30°C for 30 min. The reacted solution was added to the pore of the agarose gel plate (containing 1.5% agarose and 1.0% Birch xylan) and incubated at 30°C for 16 h, then stained with 1.0% Congo red solution for 15 min, and decolorized with 1M NaCl solution for 15 min, and finally fixed with 1M HCl solution. Halo phenomenon in agarose gel was observed.

The DNS method described by Miller (1959) was used to determine the activity of xylanase and inhibitor with some modifications. The 100 μL xylanase was added to nine centrifuge tubes (5 ml), and then different volume of inhibitory protein (0, 100, 200, 300, 400, 500, 600, 800, and 1,000 μL) was added to each tube. The xylanase-inhibitor mixture was heated at 30°C for 30 min, and ddH_2_O was added to 1,500 μL after the reaction. The 100 μL diluted enzyme-inhibitor reaction solution was mixed with 100 μL 2% Birch xylan solution. The mixture was heated at 50°C for 15 min and terminated by adding 600 μL DNS, then 5 ml water was added. The content of reducing sugar was determined by spectrophotometer at A550 nm. It was corrected by the solution of the reaction between inactivated xylanase and inactivated inhibitor. Finally, the xylanase activity was calculated according to the following formula: *E* = 1.53 × D × Y (E: xylanase activity, IU/ML; D: enzyme dilution; Y: absorbance). We assumed that the activity of xylanase in the reaction solution was 100% without addition of inhibitors. After adding inhibitors, the relative activity of xylanase in the reaction solution was calculated according to the measured value of absorbance.

### Extraction of Recombinant Protein and Inhibitory Effect of XIs on Xylanase-Induced Cell Necrosis

*Pichia pastoris* culture containing recombinant xylanases (FGSG_03624, FGSG_10999, and FGSG_11487), recombinant XIs (TAXI–IA, TAXI–IAb, TAXI–IIA, TAXI–IIAb, TAXI–III, and TAXI–IV) and pPIC9K (CK) were centrifuged at 10,000×*g* for 10 min. The supernatant was filtered successively through the filter membrane (Rasco, China) with pore sizes of 0.8, 0.45, and 0.22 μm, then the protein was precipitated overnight with 75% ammonium sulfate at 4°C. After precipitation, the precipitate was recovered at 10,000×*g* for 30 min at 4°C. The precipitate was dissolved in deionized water, and the recovered supernatant was dialyzed and desalted with a dialysis bag (YuanYe, China) at 4°C for 12 h. Finally, the protein solution obtained was stored in the refrigerator at −80°C.

The extracted xylanase was incubated with xylanase inhibitor or *P. pastoris* secretion (CK) for 10 min at 30°C. Xylanase, xylanase inhibitor, *P. pastoris* secretion (CK), xylanase-inhibitor mixture, and xylanase-CK mixture were injected into leaves of *Nicotiana benthamiana* (6–8 weeks) with 1 ml syringe without a needle, respectively. The symptoms of leaves were observed and recorded for 3–5 days after infiltration. Each infiltration experiment was repeated three times.

## Results

### Identification and Characteristics of *TaXI* Genes

The conserved TAXI_N and TAXI_C domains were used to identify the presence of *TaXI* genes in the wheat genome. We constructed the wheat-specific Hidden Markov Model (HMM) file and used TAXI-type proteins of wheat, barley, rye, and durum wheat as query sequences to search the whole wheat protein sequences using local BLASTP. A total of 287 *TaXI* candidate genes were identified in the latest wheat genome database. After excluding candidate genes with seriously incomplete and without TAXI_N or TAXI_C domain, 277 non-redundant genes were finally identified as wheat *TaXI* gene family members. The predicted genes were named based on the position of these genes on the chromosome. The information of gene name, gene ID, chromosome position, amino acid length, isoelectric point (PI), molecular weight, subcellular localization, and signal peptide were shown in Supplementary Table 1.

**FIGURE 1:**
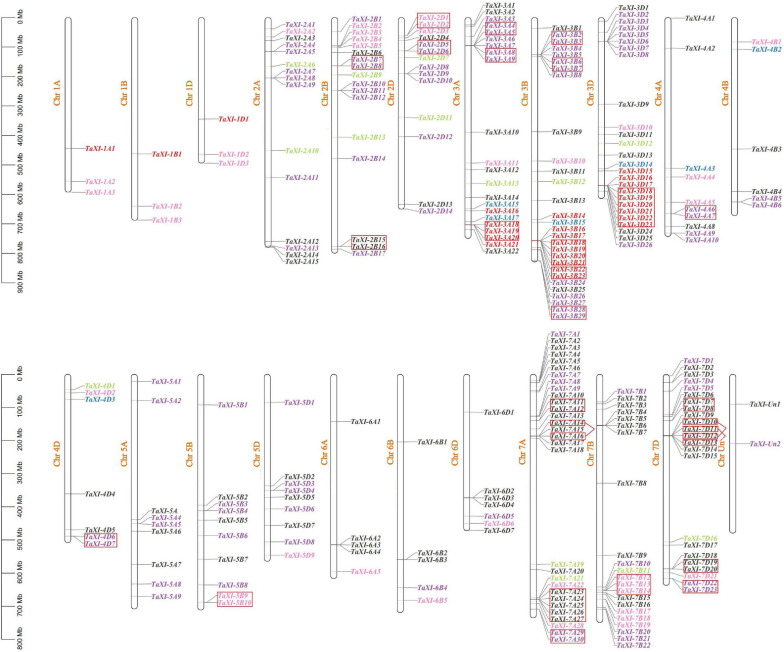
Chromosomal location and gene tandem duplication of *TaXIs*. The blue, red, pink, green, purple, and black fonts represent the subfamily *XI1*–*XI6*, respectively. The tandem duplicated genes are marked by small boxes of red color (among them, *TaXI-7A14/TaXI-7A16*, *TaXI-7D10/TaXI-7D12*, and *TaXI-7D11/TaXI-7D13* are tandem duplicated gene pairs).

Only two predicted *TaXI* genes were marked on the scaffold, others were unevenly distributed on 21 chromosomes of wheat (Figure 1). Among them, the homologous chromosome group 3 contained the most *TaXI* genes (77) with 22, 29, and 26 *TaXI* genes on 3A, 3B, and 3D, respectively, followed by the homologous chromosome group 7 with 75 *TaXI* genes, and the chromosome 7A had the highest density of *TaXI* genes with 30 on it. However, the chromosomes 1A, 1B, and 1D each contained only three *TaXI* genes. The lengths of TAXI proteins ranged from 318 to 632 amino acids, the isoelectric point varied from 4.49 to 9.98, and the molecular weight from 33.92 to 68.85 KDa (Supplementary Table 1). The results of subcellular localization showed that 61.73% TAXI family members were located in the extracellular matrix, 29.96% in the cell membrane, few members in chloroplast, mitochondria, and nucleus (Supplementary Table 1). The signal peptide analysis showed that 84.84% members had a signal peptide structure, which was composed of 16–33 amino acids (Supplementary Table 1).

### Phylogenetic Analysis and Duplication Events of Wheat *TaXI* Gene Family

**FIGURE 2:**
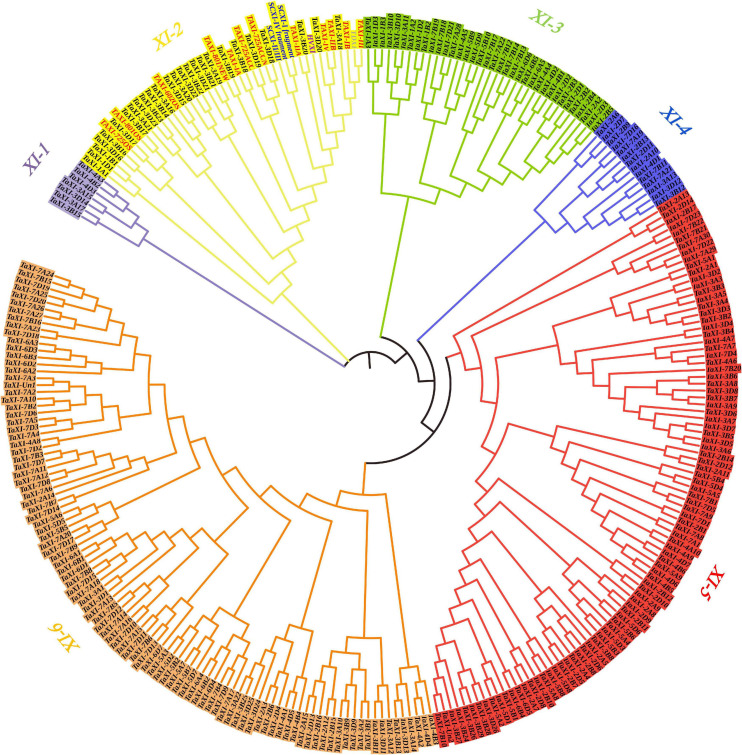
Phylogenetic tree generated from the TAXI family members in wheat, barley, rye, and durum wheat. TAXI proteins from wheat, barley, rye, durum wheat, and predicted TAXI in this study are represented in red, violet, blue, gray, and black fonts, respectively. Different colors represent different subfamilies in the *TaXI* gene family.

The NJ method was used to construct phylogenetic tree for 294 TAXI-type proteins from wheat, barley, rye, and durum wheat (Figure 2), and phylogenetic tree for 277 predicted wheat TAXI proteins (Supplementary Figures 1A, 2A). These TAXI-type proteins were divided into six subfamilies: *XI-1*, *XI-2*, *XI-3*, *XI-4*, *XI-5*, and *XI-6* (Figure 2 and Supplementary Figure 2A). The number of TAXIs in each subfamily varied from 7 members in the subfamily *XI-1* to 101 members in the subfamily *XI-6*. Interestingly, all reported TAXI-type inhibitory proteins of cereal crops, including wheat, barley, rye, and durum wheat, were grouped into the *XI-2* subfamily, indicating closely relationships and similar properties and functions among them.

To better understand the formation and the causes of expansion of wheat *TaXI* gene family, we performed homeologous group analysis for the 277 predicted wheat TAXI protein sequences to reveal the distribution of each gene on the chromosomes, and clustering analysis to reveal replication events of this gene family. Ninety-two homologous gene pairs or clusters were identified at the terminal node of the phylogenetic tree from 277 putative TAXI-type proteins (Supplementary Figure 1A). Among them, 54 gene clusters of *TaXIs* exhibited homology of 1:1:1, corresponding to *TaXIs* copy on the A, B, and D sub-genomes, which was named as triplets (Supplementary Figure 1A). However, gene losses occurred in some triplets, 38 gene pairs have copy on only two of the three homoeologous chromosomes (Supplementary Figure 1A), 39 genes were not found homologs in wheat genome. The high proportion (85.9%) of the homoeologous pairs or clusters in wheat TAXI family indicated that polyploidization might be one of the main causes resulting in the expansion of *TaXI* gene family in wheat.

**FIGURE 3:**
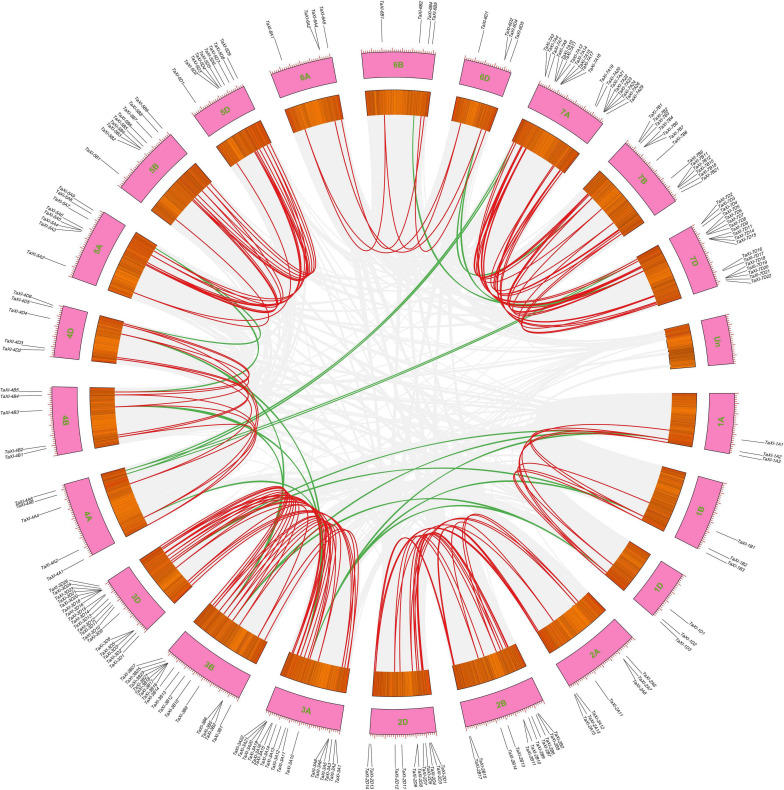
Segmental duplication gene pairs in the wheat genome. Gray lines in the background indicate the synteny blocks within the whole wheat genome, red lines denote the segmental duplication *TaXI* gene pairs between the homologous chromosomes, and green lines denote the segmental duplication *TaXI* gene pairs between the non-homologous chromosomes.

In addition to polyploidization, the tandem and segmental duplications of genes were also the main causes of the formation of wheat gene family (Cannon et al., 2004; Li et al., 2015a). A total of 63 genes (22.7%) in wheat *TaXI* gene family were identified as tandem duplication genes, which were located on chromosomes 2B, 2D, 3A, 3B, 3D, 4A, 4D, 5B, 7A, 7B, and 7D with 4,4,7,12,6,2,2,2,11,3,10 genes, respectively (Figure 1 and Supplementary Table 2). Among them, the chromosome 3D contained the largest tandem duplication gene cluster (six *TaXI* genes), followed by chromosome 7A with five *TaXI* genes. Furthermore, MCScanX was used to analyze the segmental duplication of wheat *TaXI* genes. A total of 171 pairs of *TaXI* genes (69.7%) were identified as segmental duplication (Figure 3 and Supplementary Table 2). These *TaXI* genes were distributed on all chromosomes, and the most genes (52) were located on the 3th homologous chromosome group. These results suggested that tandem and segmental duplication events are also one of causes for the expansion of the *TaXI* gene family.

The ratio of non-synonymous (Ka) to synonymous (Ks) can be used to measure selective pressure on duplication events (Akhunov et al., 2013). We calculated the Ka/Ks ratio for the tandem and segmentally duplicated *TaXI* genes, and the approximate dates of replication events (Supplementary Table 2). In tandem duplication gene pairs, the Ka and Ks values of both *TaXI-7B12* and *TaXI-7B13* were 0, only the Ka value of *TaXI-7D22*/*TaXI-7D23* was greater than the Ks value (3.5925), 0 for *TaXI-7B12/TaXI-7B14* and *TaXI-7B13/TaXI-7B14*, and 0.1424–0.8651 for other gene pairs. The Ka/Ks values of 171 segmental duplication gene pairs were 0–0.8855. The Ka/Ks values of these *TaXI* gene pairs suggested that only *TaXI-7D22*/*TaXI-7D23* suffered positive selection, the other replicated genes suffered purifying selection, except *TaXI-7B12*/*TaXI-7B13*. Duplication events in the wheat *TaXI* gene family might have occurred between 0.2278 and 81.0593 Mya (millions of years) ago (Supplementary Table 2).

### Gene Structure of *TaXIs* and Motif Composition of *TAXIs*

The diversity of gene structure promotes the evolution of multigene families, so the analysis of gene structure can provide important information about gene function, organization and evolution (Koralewski and Krutovsky, 2011; Xu et al., 2012). To better understand the structural diversity of *TaXI* genes, we generated exon/intron organization maps from the coding sequences of each *TaXI* gene (Supplementary Figure 2B). The structural analysis showed that the number of introns in the *TaXI* genes were 0,1,2,3,9,10 or 11 (Supplementary Figure 2B). Most *TaXI* genes (181) didn’t contain introns. The Supplementary Figure 2B showed that the exon/intron structures in the *TaXI* genes varied among diverse subfamilies, but were relatively conserved among the genes within the same subfamily. All genes in the *XI-4* subfamily contained 9–11 introns, but the genes in other subfamilies contained only 0–3 introns.

In addition to exon/intron structure, conserved motifs could be important to the diversified functions of TAXI proteins. Therefore, we used the MEME web server to search the conserved motifs shared among the 277 TAXI proteins. A total of 20 distinct conserved motifs were found, and named motif1–motif20 (Supplementary Figure 2C and Supplementary Table 3). Each of the putative motifs was annotated by searching Pfam. Among them, motif1, motif4, motif5, motif7, motif8, motif11, motif12, motif15, motif16, and motif18 corresponded to TAXi_N domain, motif2, motif3, motif6, motif10, motif13, motif14, motif17, and motif19 corresponded to TAXi_C domain, motif9 corresponded to TAXi_N and TAXi_C domains simultaneously, while motif20 has no function annotation (Supplementary Table 3). The length of these motifs varied from 27 to 132 amino acids, of which motif9 was the longest and motif17 was the shortest (Supplementary Table 3). As shown in the Supplementary Figure 2C, the wheat TAXI protein contained 4 - 7 motifs, most members had six motifs. Motif3 was the most common motif found in 162 TAXI proteins. It was worth noting that proteins in the same subfamily, particularly the most closely related members, generally have a similar motif composition, suggesting function similarities among these TAXI proteins. For example, all members of the *XI-1* subfamily contained motif8, motif14, and motif13. All members of the *XI-2* subfamily contained motif15 and motif19, and most members also contained motif20 and motif17. Similarly, most members of the *XI-3* subfamily contained motif16, motif14, motif3, and motif10. Motif20 and motif16 are unique to *XI-2* and *XI-3* subfamilies, respectively. Motif 9 was mainly present in *XI-5* subfamily, only two proteins (TAXI-4D1 and TAXI-2B13) in *XI-4* subfamily contained motif 9. Most members of *XI-1* and *XI-5* subfamilies generally contained motif2, while members of other subfamilies did not contain motif2. To a certain extent, these specific motifs might contribute to the functional divergence of TAXI proteins.

### *Cis-*Acting Elements in the Promoters of *TaXI* Genes

**FIGURE 4:**
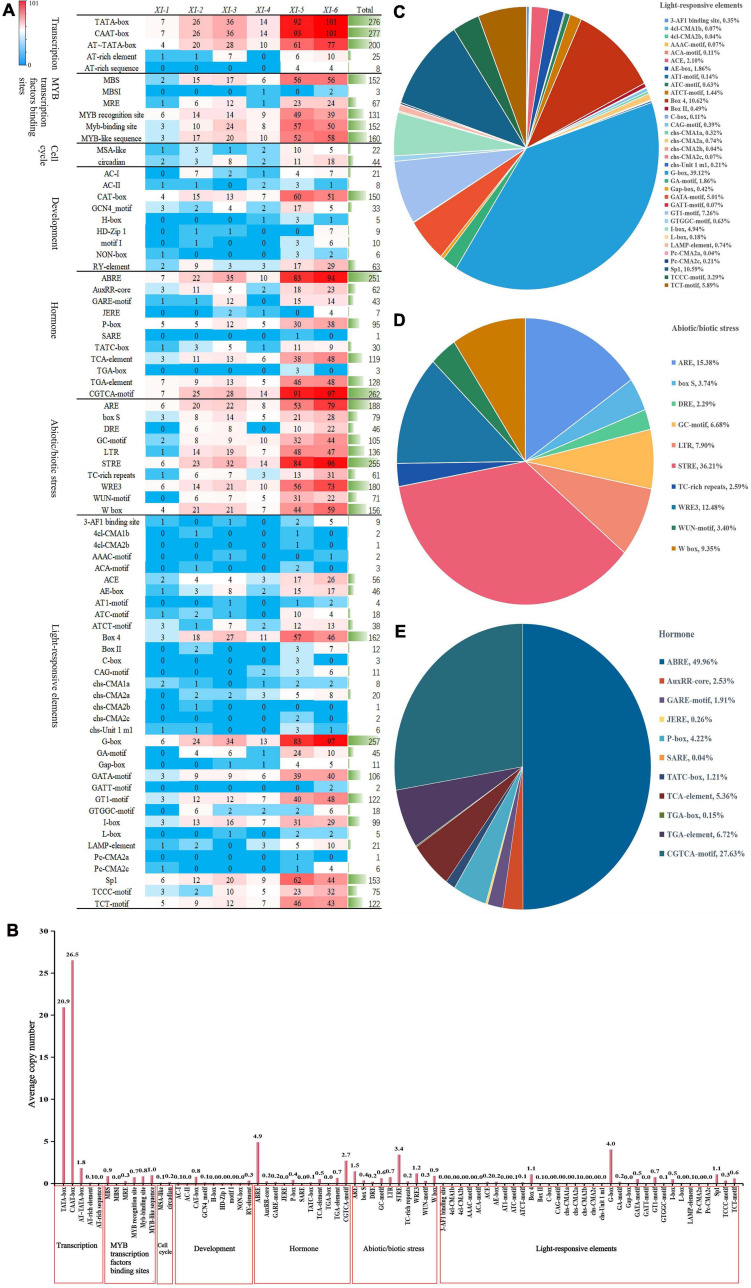
*Cis-*acting element analysis of the promoter regions of *TaXI* genes The graph was generated using *cis-*acting element names and functions of *TaXI* genes. **(A)** The number of *TaXI* genes containing different *cis-*acting elements in six different subfamilies. **(B)** Average copy number of each *cis-*acting element in the promoter regions of *TaXI* genes. **(C–E)** The number of each *cis-*acting element as a percentage of its classification.

To explore the possible biological functions of *TaXI* genes, the *cis-*acting regulatory elements were predicted for the 2.0 kb upstream promoter regions of all *TaXI* genes using PlantCARE. The predicted results showed that the promoters of *TaXI* gene family members contained the base-acting elements CAAT-box and TATA-box, except no TATA-box in the promoter of the *TaXI-3A5* gene, with an average copy number of 26.5 and 20.9, respectively (Figure 4, Supplementary Figure 1B, and Supplementary Table 5).

A large number of optical response elements were predicted in the promoter of *TaXI* gene family members, including G-box, Box-4, I-box, GT1-motif, etc., suggesting that the expression of the *TaXI* genes might be closely related to light. Among them, G-box was highly enriched in *TaXI* gene promoters, which was detected in 257 members, with an average copy number of 4 (Figure 4 and Supplementary Table 5). The promoters of most *TaXI* genes also contained a variety of *cis-*acting elements related to hormone response, such as ABRE involved in the abscisic acid (ABA) response, AuxRR-core, TGA-box, and TGA-element involved in auxin (IAA) response, GARE-motif, P-box, and TATC-box involved in gibberellin (GA) response, JERE and CGTCA-motif involved in the jasmonic acid (JA) response, and TCA-element involved in the salicylic acid (SA) response. Among them, ABRE (90.6%) and CGTCA-motif (94.6%) existed in a large number of members, with an average copy number of 4.9 and 2.7, respectively, indicating that most of the *TaXIs* might participate in JA- and ABA-mediated signaling pathways (Figure 4, Supplementary Figure 1B, and Supplementary Tables 4, 5).

A large number of *cis-*acting elements related to stress also appeared in the promoters of *TaXI* genes (Figure 4, Supplementary Figure 1B, and Supplementary Tables 4, 5). A total of 255 members had STRE element that is related to heat shock activation, osmotic stress, low PH stress, and nutritional deficiency stress, with an average copy number of 3.4. The promoters of many *TaXI* genes also contained *cis-*acting elements related to anaerobic stress (ARE and GC-motif), low-temperature response (LTR) and damage response (WRE3, WUN-motif, and W-box). This suggested that these *TaXI* genes might be involved in anoxic stress, low-temperature stress and defense injury recovery response. Besides, the promoters of some members also contained *cis-*acting elements related to tissue-specific expression and cell cycles, such as CAT-box, GCN4-motif, motifI, NON-box, RY-element, and MSA-like. Among them, CAT-box appeared in 150 *TaXI* genes, which is involved in the expression of the meristem (Figure 4, Supplementary Figure 1B, and Supplementary Tables 4, 5).

We also found that MYB transcription factor binding sites were present in the promoters of 268 *TaXI* genes (Figure 4, Supplementary Figure 1B, and Supplementary Tables 4, 5). These binding sites, mainly including MBS, MRE, MBSI, MYB recognition site, Myb-binding site, MYB-like sequence, are involved in drought-inducibility, light responsiveness, flavonoid biosynthetic genes regulation, and unknown function. In summary, we speculated that the *TaXI* gene might participate in specific signaling pathways and regulate the hormone signal transduction process and the defense response to various stresses in wheat.

### Expression Profiles of *TaXI* Genes in Various Organs and Developmental Stages

**FIGURE 5:**
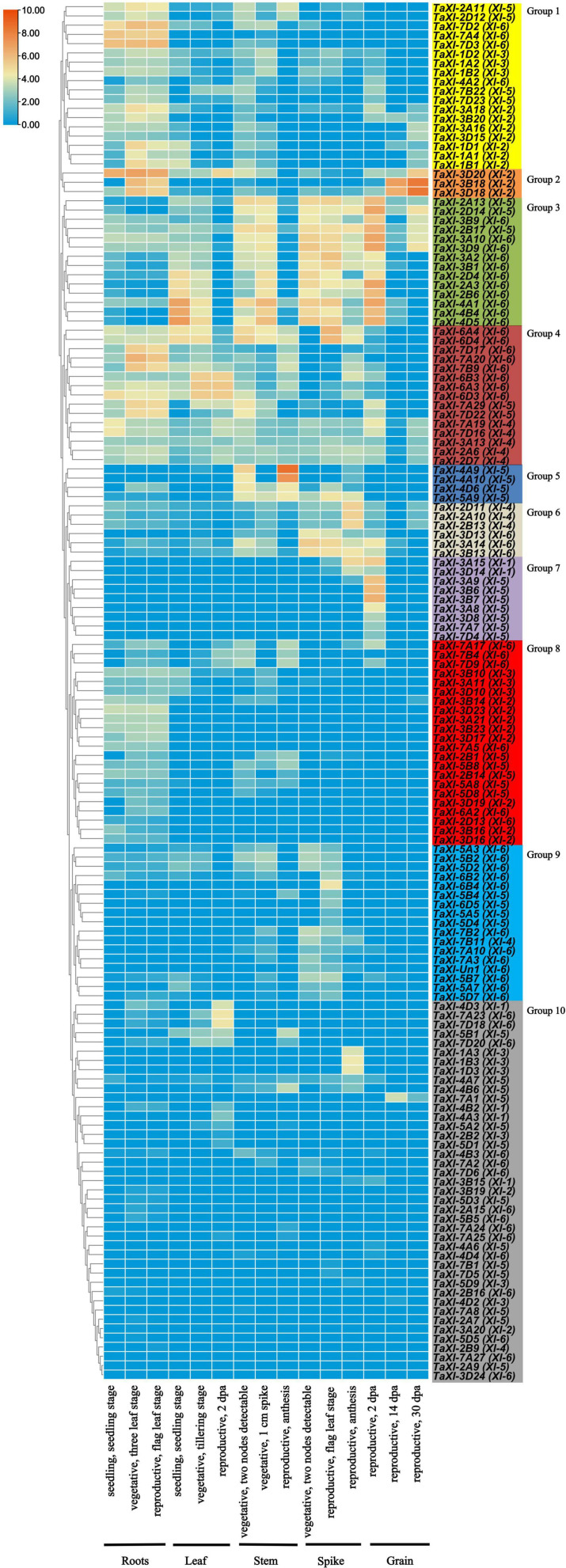
Expression profiles of wheat *TaXI* genes in five different tissues at three different developmental stages. Log_2_tpm expression values were used to create the heat map. The different colors represent the abundance of the transcripts. The *TaXI* genes were divided into 10 clusters with different expression patterns, which were indicated in different background colors.

In order to predict the roles of *TaXI* genes in growth and development, we downloaded the expression data of roots, leaves and stems at Chinese spring seedlings, vegetative growth and reproductive growth stages, as well as spikes and grains at reproductive genitalia from the WheatExp website (Clavijo et al., 2017). TBtools was used to analyze the data. Significant differences in the expression patterns of the *TaXI* genes in different tissues and developmental stages were observed (Figure 5 and Supplementary Table 6). We discarded some genes with very low or no expression (log_2_tpm = 0) (not shown in the Figure 5 and Supplementary Table 6). The remaining 149 *TaXI* genes were divided into 10 groups with different expression patterns (Figure 5). The expression of 18 genes in the group 1 in roots was higher than that in other tissues, especially *TaXI-7D2*, *TaXI-7A4*, and T*aXI7D3* which were not expressed in grains. Three genes in the group 2 were mainly expressed in roots and grains. In the group 3, 14 genes were mainly expressed in leaves, stems, spikes and grains, and the expression in leaves at seedling and vegetative growth stage was higher than that at the reproductive growth stage, and the expression in stems and spikes at vegetative growth stage was higher than that at the reproductive growth stage, the expression in grains at the early reproductive growth stage was higher than that at the middle and later stages, but the expression was very low at all stages of the root. Four genes in the group 5 were mainly expressed in stems but hardly expressed in grains. The expression of six genes in the group 6 was significantly higher in spikes than in other tissues. Nine genes in the group 7 were mainly expressed in the early growth stage of grains, especially the expression levels of *TaXI-3B6* and *TaXI-3B7* were relatively high, and these two genes were not expressed in other tissues and grains at the late growth stage. Although the genes in the group 8-group 10 were expressed in some tissues or developmental stages, the expression level was very low.

It is worth mentioning that most homologous genes have very similar expression patterns in the process of growth and development (Figure 5). In the *XI-4* subfamily, three homologous genes, *TaXI-2D11*, *TaXI-2A10*, and *TaXI-2B13*, were highly expressed in the spikes at the late reproductive growth stage, but not expressed or very low in other tissues and stages. Three homologous genes, *TaXI-3A9*, *TaXI-3B7*, and *TaXI-3D8* in the *XI-5* subfamily, were specifically expressed in early grains. Similarly, in the *XI-6* subfamily, three homologous gene clusters, *TaXI-2D4*, *TaXI-2B6*, and *TaXI-2A3* were expressed in all tissues except roots. *TaXI-6B3*, *TaXI-6A3*, and *TaXI-6D3* were mainly expressed in leaves during vegetative and reproductive stages, and *TaXI-7D17*, *TaXI-7A20*, and *TaXI-7B9* were mainly expressed in roots. However, the expression patterns of three homologous genes, *TaXI-2D7*, *TaXI-2A6*, and *TaXI-2B9* in the *XI-4* subfamily were different. The first two genes were expressed in each tissue, while the latter one was not expressed or very lowly expressed in all tissues. Similarly, *TaXI-5B1*, *TaXI-5A2*, and *TaXI-5D1* are homologous genes. But *TaXI-5B1* was expressed in leaves and stems, while *TaXI-5A2* and *TaXI-5D1* were only slightly expressed in leaves.

### Expression Profiles of *TaXI* Genes Under Abiotic Stress

In the previous analysis of the promoter of *TaXI* genes, we found that many members contain *cis-*acting elements in response to abiotic stress, such as drought, high and low temperature. Therefore, we speculated that some *TaXI* genes might play an important role in resistance to abiotic stress. To further understand the expression patterns of wheat *TaXI* genes under abiotic stress, we used the existing RNA-SEQ data to explore the expression of wheat *TaXI* genes under drought, high and low temperature stress.

**FIGURE 6:**
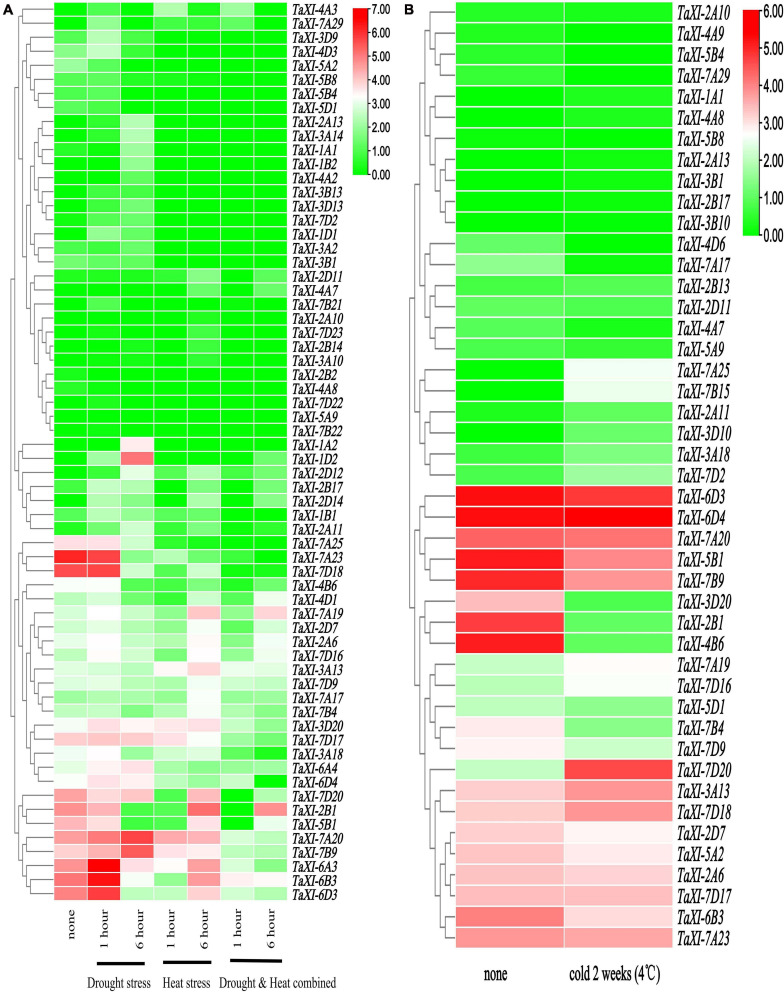
Expression profiles of *TaXI* in different abiotic stress. The heatmap was constructed using the TBtools, and the TPM values of *TaXI* genes were transformed by log_2_. The different colors represent the abundance of the transcripts. Green color represents low expression and red color indicates a high expression level (transcript abundance). **(A)** Heat map of *TaXIs* under drought and heat stress treatments. **(B)** Heat map of *TaXIs* under low-temperature stress.

The publicly available RNA-seq data of wheat leaves under drought and heat stresses were used to analyze the expression profiles of the *TaXI* genes (Figure 6A and Supplementary Table 7) (Liu et al., 2015). The heatmap (Figure 6A) showed that the expression of *TaXI-7A19*, *TaXI-2A6*, and *TaXI-7D16* in drought treatment for 1 h, high-temperature treatment for 6 h, and drought and high-temperature co-treatment for 6 h were significantly higher than those in the control group. Three homologous genes, *TaXI-6A3*, *TaXI-6B3*, and *TaXI-6D3*, were only up-regulated at 1 h of drought treatment and down-regulated under other treatments, but significantly higher in 6 h high-temperature treatment than that in 1 h high-temperature treatment. Similarly, *TaXI-1A2*, *TaXI-1D2*, and *TaXI-1B2* were up-regulated at 6 h drought treatment, especially *TaXI-1D2*, suggesting that these homologous genes had similar physiological functions. The *TaXI-7A20* and *TaXI-7B9* were slightly up-regulated or had no significant change under drought or high temperature alone, but significantly down-regulated under the co-treatment of drought and heat stresses. The expression levels of *TaXI-7A25*, *TaXI-7A23*, *TaXI-7D18*, and *TaXI-4B6* under the drought treatment for 1 h were similar to those in the control group, but decreased significantly under other treatments.

The Figure 6B and Supplementary Table 8 showed that the expression patterns of *TaXI* genes in wheat variety Manitou at the three-leaf stage after 2 weeks under low-temperature treatment (4°C) (Li et al., 2015b). The expressions of *TaXI-7A25*, *TaXI-7B15*, *TaXI-7A19*, *TaXI-7D16*, *TaXI-7D20*, *TaXI-3A13*, and *TaXI-7D18* were significantly increased under cold stress. However, the expressions of *TaXI-5B1*, *TaXI-7B9*, *TaXI-3D20*, *TaXI-2B1*, *TaXI-4B6*, *TaXI-7B4*, and *TaXI-7D9* genes were decreased under low temperature treatment. In addition, some *TaXI* genes showed low expression in both control and stress treatments. Interestingly, some homologous genes exhibited different expression patterns. For example, *TaXI-3D20* was significantly decreased under cold stress, while *TaXI-3A18* was slightly increased under low temperature treatment, *TaXI-3B20* did not express in both the cold treatment and control groups (not shown in Figure 6B and Supplementary Table 8). It suggested that the functional differentiation has been occurred in these homologous genes, and they were involved in regulating different stress signaling pathways.

### Expression Profiles of *TaXI* Genes Under Biotic Stress

All TAXI-type proteins contain TAXi_N and TAXi_C domains, both of which are related to plant resistance to pathogens (Fierens et al., 2003; Pollet et al., 2009; Moschetti et al., 2013), so we speculated that the *TaXI* gene might be involved in plant defense against pathogens. To explore the expression patterns of *TaXI* genes in wheat infected by powdery mildew, stripe rust and *F. graminearum*, we analyzed the related RNA-SEQ data on WheatExp.

**FIGURE 7:**
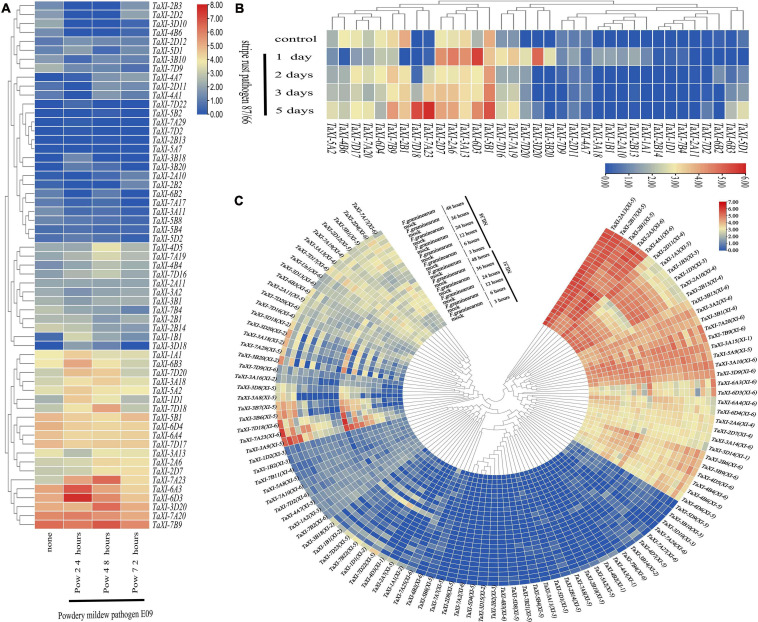
Expression profiles of *TaXI* in different biotic stress. TPM values of *TaXI* genes were transformed by log_2_ and the heatmap was constructed by TBtools. The different colors represent the abundance of the transcripts. **(A)** Heat map of *TaXIs* under powdery mildew inoculation. **(B)** Heat map of *TaXIs* under stripe rust inoculation. **(C)** Heat map of *TaXIs* under *Fusarium graminearum* inoculation.

Figure 7A and Supplementary Table 9 showed that the expression of the *TaXI* genes in wheat variety N9134 after being uninfected and infected by powdery mildew for 24, 48, and 72 h (Zhang et al., 2014). The expression of some genes increased at first and then decreased under powdery mildew infection, such as *TaXI-1A1*, *TaXI-6B3*, *TaXI-7D20*, and *TaXI-3A1*8. Compared with the control, the expression of these genes increased significantly at 24 or 48 h after inoculation, but decreased to the level of the control group after inoculation for 72 h. However, some genes, such as *TaXI-3A13*, *TaXI-3A2*, *TaXI-3B1*, and *TaXI-7B4*, were down-regulated after inoculation with powdery mildew.

The RNA-SEQ data of wheat variety Avocet (*Yr5*) infected by stripe rust was used to analyze the expression of *TaXI* genes (Figure 7B and Supplementary Table 10) (Dobon et al., 2016). The expression of some genes significantly increased in the early stage of inoculation (1 day), but decreased in the late stage of inoculation (2–5 days), such as *TaXI-3B20*, *TaXI-3D20*, and *TaXI-7D20*. *TaXI-7A23* and *TaXI-7D18* almost did not express in the control and 1 day infection, but up-regulated in the late stage of inoculation (2–5 days). After infected by stripe rust, the expression of some genes increased at first, then decreased, and then increased again, such as *TaXI-6D3*, *TaXI-3A13*, *TaXI-2A6*, and *TaXI-2D7*. The expression of *TaXI-7B9*, *TaXI-6D4*, *TaXI-7D17*, and *TaXI-4B6* in uninfected leaves was relatively high, but was down-regulated at 1 day after inoculation with stripe rust, and up-regulated at late inoculation stage (2–5 days) compared with that at 1 day after inoculation.

RNA-SEQ data was used to study the roles of *TaXI* genes in response to inoculation with *F. graminearum* for 3, 6, 12, 24, 36, and 48 h (Figure 7C and Supplementary Table 11) (Schweiger et al., 2016). The expression patterns of *TaXI* genes between the two lines, NIL51 and NIL38, were similar under the same treatment conditions. Without *F. graminearum* infection, *TaXI-3B20*, *TaXI-7A29*, *TaXI-3A18*, *TaXI-3D20*, *TaXI-3D18*, *TaXI-7D22*, *TaXI-1D1*, *TaXI-7B22*, *TaXI-7D23*, *TaXI-1B1*, and *TaXI-3B18* were almost unexpressed or very low expressed in spikes at all stages. When wheat spikes were infected by *F. graminearum*, the expression of these genes significantly increased and reached the highest level at 48 h. The expression levels of *TaXI-6A3* and *TaXI-6D3* were similar in spikes without inoculation or 3 h inoculation, and were higher than those in other periods without inoculation, but significantly up-regulated at 24 h after inoculation with *F. graminearum* and reached the highest at 48 h. The expression of *TaXI-7A20* and *TaXI-7B9* was relatively high in each stage of uninoculated spikes, and up-regulated after inoculation with *F. graminearum*. Thus, the above genes might play an important role in wheat response to *F. graminearum* infection. However, the expression of *TaXI-2B6*, *TaXI-3B9*, *TaXI-4D5*, *TaXI-4B4*, *TaXI-4B6*, and *TaXI-4D6* slightly decreased after inoculation with *F. graminearum*.

### Cloning and Characterization of Wheat *TaXI* Genes and *F. graminearum* Endo-Xylanase Genes

**FIGURE 8:**
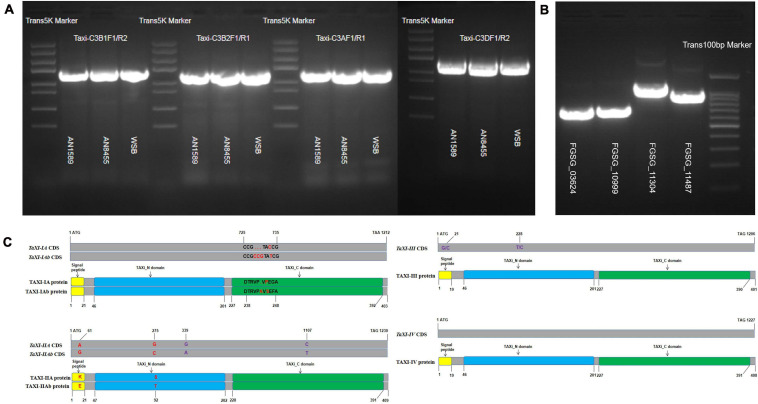
PCR product and sequence alignment. **(A,B)** PCR amplification of *TaXI* genes from three wheat varieties and *Fusarium graminearum* xylanase genes. **(C)** Sequence alignment of *TaXI* genes and proteins. The red font indicates non-synonymous mutations or inserts/deletion, and the purple font indicates synonymous mutations. The yellow rectangle represents the signal peptide, the blue rectangle represents the TAXI_N domain, and the green rectangle represents the TAXI_C domain.

Using the specific primers (Supplementary Table 12) designed by us, we amplified the gene sequences and the complete CDS sequences of *TaXI* genes from the genomic DNA and the cDNA of wheat spikes of three whet cultivars, Wangshuibai (WSB), Annong 1589 (AN1589), and Annong 8455 (AN8455) infected with *F. graminearum*. The sequences showed that none of six different xylanase inhibitor genes isolated from three wheat varieties contained introns (Figure 8A). The gene sequences amplified by primers Taxi-C3B1F1/R2 from the genomic DNA of WSB and AN1589 were consistent with the reported *TaXI–IA* and encoded TAXI–IA protein. Compared with the *TaXI-IA* gene, the gene sequence amplified from the genomic DNA of AN8455 with these primers had three base insertions and one SNP difference (Figure 8C). This new gene was named *TaXI-IAb*, and the encoded protein was named TAXI–IAb. The gene sequence amplified by primers Taxi-C3AF1/R1 from the genomic DNA of WSB was the same to the reported *TaXI–III*, which encoded TAXI–III protein. The gene sequences amplified by these primers from the genomic DNA of AN1589 and AN8455 were the same, but there were two SNP loci differences compared with the reported *TaXI–III* gene. However, they were synonymous mutations, and this gene also encoded the TAXI–III protein (Figure 8C). Similarly, the gene sequence amplified by primers Taxi-CB2F1/R1 from the genomic DNA of WSB was the same to the reported *TaXI–IIA*, which encoded TAXI–IIA protein. The PCR products amplified from the genomic DNA of AN1589 and AN8455 by these primers were consistent, but there were 4 SNP loci differences compared with the *TaXI–IIA* gene, two of which were non-synonymous mutations and the other two were synonymous mutations (Figure 8C), this new gene was named *TaXI–IIAb*, and the corresponding protein was named TAXI–IIAb. Finally, the gene sequences amplified from the genomic DNA of WSB, AN1589 and AN8455 by primers Taxi-C3DF1/R1 were completely consistent with the reported *TaXI–IV* gene sequences, encoded the TAXI–IV protein (Figure 8C).

The complete CDS sequences of xylanase genes (*FGSG_03624*, *FGSG_10999*, *FGSG_11304*, and *FGSG_11487*) were amplified from cDNA of wheat spikes inoculated with *F. graminearum* for 72 h by specific primers (Supplementary Table 12). The results showed that the coding regions of the four xylanase genes consisted of open reading frames of 687, 696, 1,146, and 984 bp, encoding proteins with 228,231,381 and 327 amino acids, respectively (Figure 8B). Some SNP loci differences between the cloned xylanase and the reported xylanase sequences were observed, but the SNP locus in *FGSG_03624*, *FGSG_10999*, and *FGSG_11487* sequences were all synonymous mutations, and did not change the encoded protein. However, four mutant bases in the *FGSG_11304* sequence changed the encoded protein, but it still belonged to the GH10 family of xylanases.

### *Triticum aestivum* Xylanase Inhibitor Genes Expression Following *F. graminearum* Infection in Wheat

**FIGURE 9:**
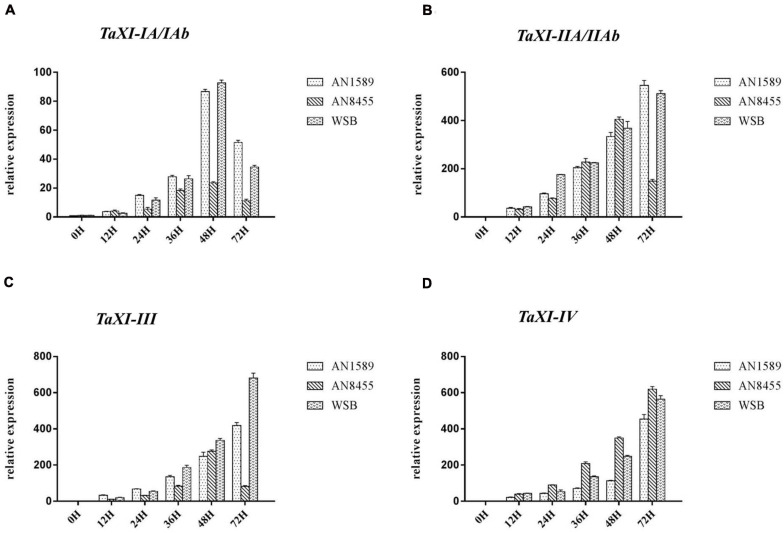
Expression patterns of *TaXIs* induced by *Fusarium graminearum* in three wheat varieties (WSB, AN1589, and AN8455). The expression level was calculated by the comparative CT method with the wheat Actin gene as the endogenous reference for normalization. **(A)** *TaXI–IA/IAb*; **(B)** *TaXI–IIA/IIAb*; **(C)** *TaXI–III*; and **(D)** *TaXI–IV*.

We used qRT-PCR to detect the expression changes of six *TaXI* genes cloned in this study, to explore whether these genes play a role in wheat resistance to *F. graminearum* infection (Figure 9). The results showed that the expressions of these six *TaXI* genes were up-regulated in all tested wheat varieties under *F. graminearum* inoculation. However, the expression increase of *TaXI–IA/IAb* was much lower than that of *TaXI–IIA/IIAb*, *TaXI–III*, and *TaXI–IV* under *F. graminearum* infection (Figure 9). The expression of *TaXI–IA/IAb* was up-regulated and then down-regulated in all three varieties, and reached the maximum at 48 h after infected by *F. graminearum*. The expression level of *TaXI–IA/IAb* was 79.78 times (WSB), 97.36 times (AN1589), and 20.97 times (AN8455) higher than that in the respective control group at 48 h (Figure 9A). Within 72 h after inoculation with *F. graminearum*, the expression of *TaXI–IIA* and *TaXI–III* genes increased continuously in WSB and AN1589, and reached the peak at 72 h. The expression level was 404.84 times (*TaXI–IIA*, WSB), 376.87 times (*TaXI–IIA*, AN1589), 673.69 times (*TaXI–III*, WSB), 323.84 times (*TaXI–III*, AN1589) higher than those in their respective control (Figures 9B,C). However, the expression of *TaXI–IIAb* and *TaXI–III* genes increased at first and then decreased, and reached the peak at 48 h in AN8455, the expression level was 381.87 times (*TaXI–IIA*) and 291.89 times (*TaXI–III*) higher than those in their respective control (Figures 9B,C). The expression of *TaXI–IV* gene increased continuously in all three wheat varieties and reached the maximum within 72 h, which was up-regulated by 523.09 times (WSB), 382.44 times (AN1589), and 596.35 times (AN8455) compared with their respective control (Figure 9D). These results suggested that the expression of six *TaXI* genes cloned in this study were induced by *F. graminearum*, and they might involve in the process of wheat resistance to *F. graminearum* infection and play a positive role.

### Dual Role of TAXI-Type Xylanase Inhibitors in Wheat Resistance to *F. graminearum* Infection

We cloned the mature coding region of four xylanase genes and six XI genes into the pPIC9K vector to obtain the pPIC9K–*FGSGs* and pPIC9K–*TaXIs* plasmid by the method of homologous recombination. Then we transformed the recombinant plasmids into *P. pastoris* cells, respectively. Finally, we used the XIs and xylanases that were heterologously expressed in *P. pastoris* cells to carry out the following experiments.

**FIGURE 10:**
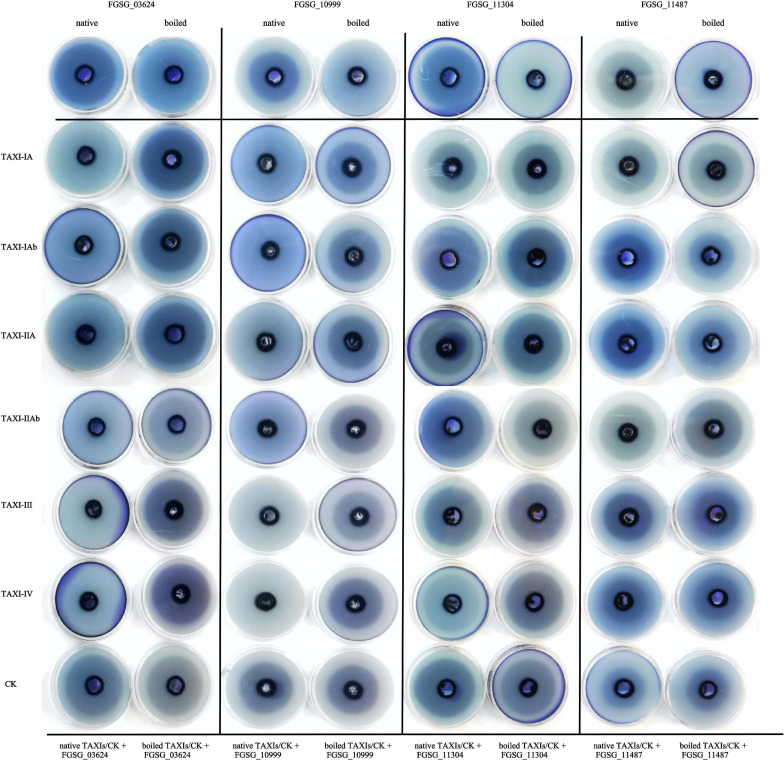
Agarose diffusion assay. The extracted crude enzyme solution of xylanases (native or boiled, 100 μL) and xylanase inhibitors (native or boiled, 600 μL) were added to the agarose gel plate, which contains 1% birch xylan. The halo indicates that xylanase activity is not inhibited. No halo indicates inactivity of xylanase.

The extracted crude enzyme solution of xylanase and xylanase inhibitor were added to the agarose gel plate, which contains 1% birch xylan, for agarose gel diffusion experiment (Figure 10). When only xylanase crude protein was added, the agarose gel plate produced a halo phenomenon. However, only with the addition of boiled inactivated xylanase crude protein, the agarose gel did not appear halo phenomenon. The results showed that all the four xylanases expressed in *P. pastoris* could degrade xylan. When the xylanase crude protein from either FGSG_03624 or FGSG_10999 was added to the agarose gels with six different xylanase inhibitor crude proteins, respectively, the agarose gels showed no halo. While halo phenomenon appeared when inactivated inhibitors and either FGSG_03624 or FGSG_10999 were added to the agarose gel. The results showed that six XIs heterogeneously expressed in *P. pastoris* could inhibit the activities of xylanase FGSG_03624 and FGSG_10999. Halo phenomenon appeared when either natural inhibitor or inactivated inhibitor was added into agarose gel with the crude protein of xylanase FGSG _ 11304 or FGSG _ 11487, suggesting that the six XIs had no inhibitory activity on xylanase FGSG_11304 and FGSG_11487.

**FIGURE 11:**
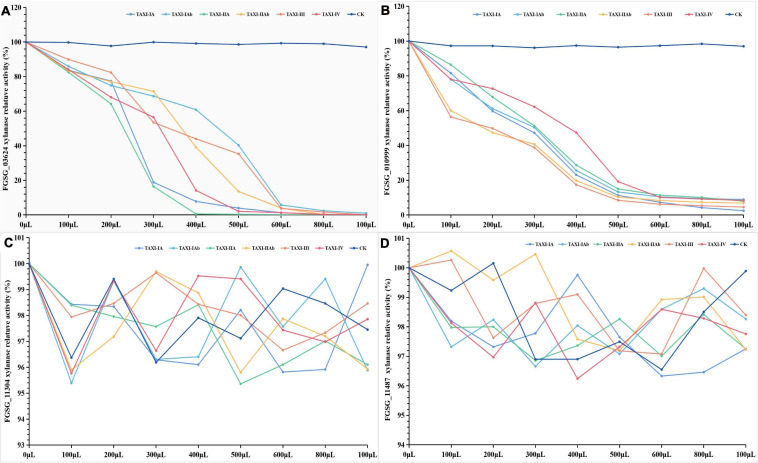
Inhibition activities of six *TAXI*-type inhibitors crude protein extracted from *Pichia pastoris* culture medium against four *Fusarium graminearum* xylanass activities. The different colored lines represent different TAXI inhibitors. FGSG_03624 **(A)**, FGSG_10999 **(B)**, FGSG_11304 **(C)**, and FGSG_11487 **(D)**. Among them, **(C,D)** show that the relative activity of FGSG_11304 (95.36–100%) and FGSG_11487 (96.24–100.57%) xylanase did not change significantly with the increase of six *TAXI* inhibitors doses, indicating that the activity of xylanase was not inhibited by the six inhibitors.

In addition, we also conducted DNS assay to study the inhibition properties of TAXI-type xylanase inhibitors against the xylanase activity of *F. graminearum.* It did not significantly change the relative activity of xylanase FGSG_03624 (97.12–99.93%), FGSG_10999 (96.16–98.40%), FGSG_11304 (96.19–99.41%) or FGSG_11487 (96.55–100.65%) and the color of the chromogenic solution when the secretion (CK) of *P. pastoris* was mixed with these xylanases, respectively (Figure 11, Supplementary Figures 3A–D, and Supplementary Table 13). When the six inhibitors were each mixed with the xylanase FGSG_03624 or FGSG_10999, the relative activity of xylanase FGSG_03624 or FGSG_10999 decreased and the color of the chromogenic solution gradually faded with the increase of the dose of inhibitor (Figures 11A,B, Supplementary Figures 3E–J, Supplementary Table 13). When the inhibitor dose was six times that of xylanase, the relative activity of FGSG_03624 xylanase dropped rapidly to less than 6%, and that of FGSG_1099 xylanase dropped to less than 12%. The xylanase activity of FGSG_03624 was almost completely inhibited when the amount of inhibitor was increased to 1 mL (<1.0%), while the relative activity of FGSG_1099 xylanase decreased to less than 10%. However, when the crude protein of the six inhibitors were each mixed with the xylanase FGSG_11304 or FGSG_11487, the relative activity of xylanase FGSG_11304 (95.36–100%) or FGSG_11487 (96.24–100.57%) did not change significantly, and the color of the chromogenic solution did not change with the increase of the dose of inhibitors (Figures 11C,D, Supplementary Figures 3K–P, and Supplementary Table 13). The results showed that the six heterologous XIs could inhibit the activities of xylanase FGSG_03624 and FGSG_10999, but had no inhibitory effect on xylanase FGSG_11304 and FGSG_11487.

**FIGURE 12:**
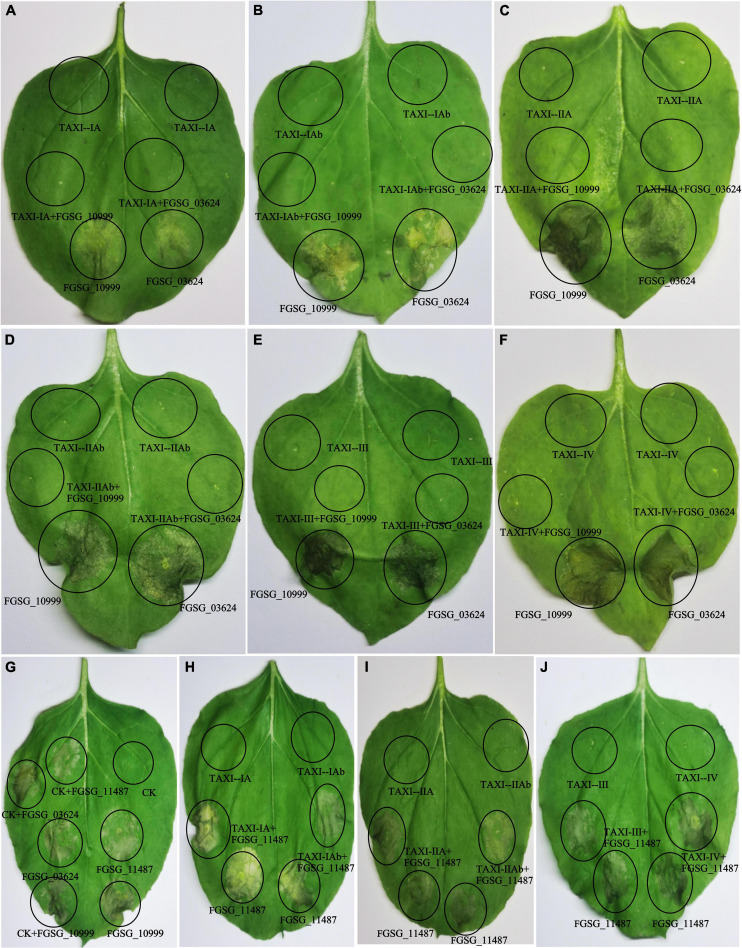
Suppression of xylanase-triggered cell death by xylanase inhibitors. Xylanase, xylanase inhibitor, *P. pastoris* secretion (CK), xylanase-inhibitor mixture, and xylanase-CK mixture, which were extracted from *P. pastoris* culture medium, were injected into leaves of *Nicotiana benthamiana* (6–8 weeks). The phenotype of cell death was scored and photographed at 3–5 days after infiltration. **(A)** TAXI-IA, FGSG_03624, and FGSG_10999 were injected separately or together into tobacco leaves; **(B)** TAXI-IAb, FGSG_03624, and FGSG_10999 were injected separately or together into tobacco leaves; **(C)** TAXI-IIA, FGSG_03624, and FGSG_10999 were injected separately or together into tobacco leaves; **(D)** TAXI-IIAb, FGSG_03624, and FGSG_10999 were injected separately or together into tobacco leaves; **(E)** TAXI-III, FGSG_03624, and FGSG_10999 were injected separately or together into tobacco leaves; **(F)** TAXI-IV, FGSG_03624, and FGSG_10999 were injected separately or together into tobacco leaves; **(G)** CK, FGSG_03624, FGSG_10999 and FGSG_11487 were injected separately or together into tobacco leaves; **(H)** TAXI-IA, TAXI-IAb, and FGSG_11487 were injected separately or together into tobacco leaves; **(I)** TAXI-IIA, TAXI-IIAb, and FGSG_11487 were injected separately or together into tobacco leaves; **(J)** TAXI-III, TAXI-IV, and FGSG_11487 were injected separately or together into tobacco leaves.

To investigate whether XIs can inhibit plant cell death induced by xylanase, we used syringes to permeate the extracted inhibitors and xylanases into leaves of *Nicotiana benthamiana*. When we only injected XIs (TAXI–IA, TAXI–IAb, TAXI–IIA, TAXI–IIAb, TAXI–III or TAXI–IV) or secretions (CK) of *P. pastoris*, it did not cause cell death (Figure 12). Cell necrosis was found in areas infiltrated by xylanase (FGSG_03624, FGSG_10999 or FGSG_11487) alone and these xylanases together with CK (Figure 12G). In addition, six XIs each co-infiltrated with xylanase FGSG_11487 also induced cell necrosis (Figures 12H,I,J). However, when six XIs each infiltrated the leaves with xylanase FGSG_03624 or FGSG_10999, no obvious cell necrosis in the infiltrated area was observed (Figures 12A–F). The results showed that all the six XIs could inhibit the cell necrosis induced by xylanase FGSG_03624 or FGSG_10999, but had no inhibitory effect on FGSG_11487.

## Discussion

### Identification of *TaXI* Gene Family

Many studies have shown that TAXI-type inhibitors are the class of inhibitory proteins produced by plants to reduce the activity of xylanase in pathogens (Gebruers et al., 2001; Belien et al., 2005; Moschetti et al., 2013). Successively, many TAXI-type inhibitors were isolated from wheat (TAXI–IA, -IB, -IIA, -IIB, -III, and -IV), barley (HVXI), rye (SCXI-I-IV) and durum wheat (TDXI) (Debyser et al., 1997, 1999; Fierens et al., 2003; Goesaert et al., 2003, 2004; Igawa et al., 2004; Raedschelders et al., 2005). So we speculated that *TaXI*-type genes most likely occur as multigene families in cereals. However, there has been no comprehensive study of the *TaXI* gene family in wheat.

With the publication of wheat reference genome data, it is possible to identify a gene family at the whole genome level. To further explore the function of the wheat *TaXI* genes, we searched the wheat reference genome and found that a total of 277 wheat *TaXI* genes that were clustered into six subfamilies (named as sub. *XI1*-*XI6*). It was consistent with TAXI-type inhibitors that have been reported, many predicted TAXI inhibitors (84.84%) have signal peptide structure, and most of them (91.69%) were predicted to locate in the extracellular matrix or plasma membrane. The signal peptide structure and apoplast/cytomembrane localization of TAXI inhibitors may be helpful for them to inhibit the activity of xylanase secreted by pathogens.

### Chromosome Localization and Duplication of *TaXIs* in Wheat

The predicted 277 *TaXI* genes were unevenly distributed on 21 chromosomes of wheat, a large number of *TaXI* genes were concentrated on the third and seventh homologous chromosome groups. However, the *TaXI* genes are evenly distributed on A, B, and D subgenomes, containing 94, 92, and 89 genes, respectively. This suggests no significant change in the content of the *TaXI* gene in wheat A, B, and D subgenomes, and no obvious preference for the retention and loss of homologous genes on the subgenomes during the two natural hybridizations and doubling of wheat.

Recent studies have showed that about 70–80% of angiosperms have undergone duplication events (Blanc et al., 2003; Bowers et al., 2003; Paterson et al., 2004). Wheat is an allohexaploid species with a complex genetic basis, which was evolved from three diploid species through two natural hybridizations (Feldman and Levy, 2005). Therefore, most genes in wheat usually have three homoeologous sites (Feldman and Levy, 2005; Ling et al., 2013). Based on the method described by Wang et al. (2016), we identified 54 homoeologous gene clusters with one copy each on A, B, and D homoeologous chromosomes for the wheat *TaXI* gene family. These genes accounted for 58.5% of the *TaXI* gene family, which was significantly higher than the proportion of homoeologous triplets in the whole wheat genome (35.8%) (Appels et al., 2018), indicating that polyploidization of wheat is the main reason for the expansion of the *TaXI* gene family. Inversion and crossover might occur between homoeologous chromosomes during the evolution of wheat, which would lead to the division of a coding region or the deletion of some homoeologous sequences, thus leading to the phenomenon of partial homoeologous copy loss (Lynch and Force, 2000). The homologous genes loss also exists in the wheat *TaXI* gene family. We identified 38 homologous gene pairs that contained two copies on the A, B, or D homoeologous chromosome. Also, most of these homologous genes were clustered on the third and seventh homoeologous chromosome groups, which was consistent with the analysis of gene localization on the chromosomes.

Tandem duplication and segmental duplication are widely found in plant genomes, and also the main reasons for the formation of gene families (Akhunov et al., 2013; Li et al., 2015a). We found 63 genes in the wheat *TaXI* gene family had tandem duplication, and each group of tandem duplication contained 2–6 genes. Additionally, we also identified that 193 genes had segmental duplication, which were distributed on all chromosomes. We found that these tandem duplication and segmental duplication genes are gathered on the group 3 and 7 chromosomes, which was in line with the above analysis of chromosome localization. The Ka/Ks ratios of these replicated gene pairs were less than 1, except *TaXI-7B12/TaXI-7B13* and *TaXI-7D22*/*TaXI-7D23*, indicating that most *TaXI* genes have undergone negative selection in wheat. Gene duplication events might have occurred about 0.2278–81.0593 million years ago.

### Inference of *TaXIs* Function Based on Gene Structure, Protein Conserved Motif, Promoter *Cis-*Acting Element and Expression Profiles

The gene structure can be used as supporting evidence to determine the evolutionary relationship between genes or species, and the distribution of motifs among proteins is indicative of evolutionary relationship as deduced by phylogenetic tree, so they both play important roles in the evolution and function of gene families (Koralewski and Krutovsky, 2011; Xu et al., 2012; Gupta et al., 2014). In general, according to the phylogenetic analysis, the wheat *TaXI* genes within the same subfamily shared similar exon-intron structures and motif compositions, suggesting that they might have similar properties and functions, but differences were present among different subfamilies. We analyzed the exon/intron structure of 277 identified *TaXI* genes. Most members of the *TaXI* gene family (65.34%) did not contain introns, corresponding well with previous studies on *TaXI* genes (Fierens et al., 2003; Igawa et al., 2004; Raedschelders et al., 2005). *TaXI* genes within the same subfamily also appeared to have different exon/intron structures. For example, six genes in the *XI-3* subfamily contain one intron, while other genes have no intron. This might be due to the influence of selection pressure on the formation of wheat gene structure and evolution, which leads to gene differentiation, and then evolves into different exon/intron structures and performs different functions (Altenhoff et al., 2012). Among the 20 motifs identified from 277 TAXI protein sequences, the motifs except motif 20 correspond to the TAXi_N domain or TAXi_C domain, which are specific to TAXI-type inhibitors and are related to degrading the xylanase activity of pathogens (Fierens et al., 2003; Pollet et al., 2009; Moschetti et al., 2013). It indicated that some TAXI proteins might be related to plant disease resistance.

Promoters play a key role in regulating the spatio-temporal expression and expression efficiency of genes (Kong et al., 2012). Igawa et al. (2004) cloned and analyzed the upstream promoter region of *TaXI–III* gene, and found several common sequences of *cis-*acting elements involved in pathogen- and wound-inducible gene expression, including GCCbox, Wbox, AS-1 and type I Myb-binding sites, in the promoter region. These *cis-*acting elements were often found in promoters of pathogenesis-related (PR) genes and defense-related genes with wound inducibility (Rushton and Somssich, 1998), and they are also consensus binding sequences of major transcription factors (TFs) that have roles in plant defense, including ERF, WRKY, bZIP (basic domain/Leu zipper) TF family TGA and Myb1 (Redman et al., 2002; Dong et al., 2003; Ferrari et al., 2003; Thurow et al., 2005). The *cis-*acting elements related to the defense against pathogens were also widely found in the identified 277 *TaXI* genes, so the expression of some genes could be induced by pathogenic microorganism. We found that the expression levels of *TaXI-3B20*, *TaXI-7A29*, *TaXI-3A18*, *TaXI-3D20*, *TaXI-3D18*, *TaXI-7D22*, *TaXI-1D1*, *TaXI-7B22*, *TaXI-7D23*, *TaXI-1B1*, and *TaXI-3B18* significantly increased after being infected by *F. graminearum*, the expression of *TaXI-1A1*, *TaXI-6B3*, *TaXI-7D20*, and *TaXI-3A18* was induced by powdery mildew, and the expression of *TaXI-3B20*, *TaXI-3D20*,*TaXI-7D20*,*TaXI-7A23*, and *TaXI-7D18* was induced by stripe rust, according to transcriptome data (Zhang et al., 2014; Dobon et al., 2016; Schweiger et al., 2016). All of these genes contained *cis-*acting elements involved in plant disease resistance. We also analyzed the expression of six *TaXI* genes cloned in this study in three wheat varieties infected by *F. graminearum*, the results showed that these genes were up-regulated after inoculation with *F. graminearum*, which was consistent with the results of transcriptome data (Schweiger et al., 2016; Igawa et al., 2004).

Moreover, the promoters of most *TaXI* genes also have many other types of *cis-*acting elements related to plant hormones, including MeJA, ABA, SA, GA, IAA, and ET, indicating that *TaXI* genes might play important roles in plant hormone pathways. The expression analysis of wheat leaves sprayed with exogenous salicylic acid and methyl jasmonate showed that the expression of *TaXI–I* and *TaXI–III/IV* couldn’t be induced by exogenous salicylic acid, but could be induced by exogenous methyl jasmonate, and the expression of *TaXI–III/IV* increased more significantly than *TaXI–I* (Igawa et al., 2005). This result is due to the fact that their promoters contain elements related to jasmonate (CGTCA-motif) but not salicylic acid.

A large number of elements related to environmental stress were also present in the promoter region of *TaXI* genes, which suggested that these genes might be regulated by abiotic stress, such as drought, high temperature, and low temperature. In this study, transcriptome data were used to explore the expression patterns of the *TaXI* genes under drought, high temperature and low-temperature stress (Pollet et al., 2009; Li et al., 2015b). It was found that there were great differences in the expression patterns of *TaXIs* under different treatment conditions. Some genes were up-regulated under more than one stress, such as *TaXI-7A19* and *TaXI-7D16. TaXI-1A2* was specifically expressed under drought stress and *TaXI-7B15* was specifically expressed under low-temperature stress. These *TaXI* genes all contained the STRE elements. These abiotic stress-induced *TaXI* genes provide valuable information for further revealing the role of *TaXI* in wheat response to stress.

We also analyzed the expression patterns of *TaXI* genes in various organs and developmental stages using public transcriptome data (Clavijo et al., 2017). The results were consistent with the results of Igawa et al. (2004), the expression profiles of the wheat *TaXI* genes were different in different tissues and different developmental stages. Some *TaXI* genes even showed tissue-specific expression patterns. For example, seven *TaXI* genes were mainly expressed in early grains, including *TaXI-3A9*, *TaXI-3B6*, *TaXI-3B7*, *TaXI-3A8*, *TaXI-3D8*, *TaXI-7A7*, and *TaXI-7D4*, which all belong to the *XI-5* subfamily. *TaXI-4A9* and *TaXI-4A10* were mainly expressed in stem. And 18 genes in the group 1 were mainly expressed in roots. In general, homologous genes tend to have similar expression patterns, so they retain similar physiological functions in the evolution of wheat. However, there are differences in the expression patterns of some homologous genes, such as *TaXI-2D7*, *TaXI-2A6* and *TaXI-2B9*, *TaXI-5B1*, *TaXI-5A2* and *TaXI-5D1*, which indicated that some homologous genes might lose their function or gain new functions after polyploidization in wheat evolution (Wang et al., 2016).

### Dual Function of TAXI-Type Xylanase Inhibitors

Expression of some *TaXI* genes was induced by *F. graminearum*, suggesting that these TAXI-type XIs might be involved in the defense function of plants. It has previously been demonstrated that wheat TAXI–IA inhibitor could inhibit the activity of endoxylanases [XylA (FGSG_10999) and XylB (FGSG_03624)] from *F. graminearum* (Belien et al., 2005). It was found that the crude protein and purified protein of TAXI–III extracted from transgenic durum wheat also inhibited the activity of xylanase FGSG_10999 and FGSG_03624 (Moschetti et al., 2013). In this study, besides TAXI–IA and TAXI–III, we found that the proteins encoded by four other xylanase inhibitor genes (*TaXI–IAb*, *TaXI–IIA*, *TaXI–IIAb*, and *TaXI–IV*) also inhibited the activity of xylanase FGSG_10999 and FGSG_03624, but had no inhibitory activity on xylanase FGSG_11304 and FGSG_11487 of GH10 family.

Xylanase inhibitors play a dual role in the resistance to pathogen infection. Some studies have also shown that TAXI-type inhibitors can not only restrain the xylanase activity of pathogens from degrading xylan of the plant cell wall, but also inhibit the plant cell death caused by xylanase (Belien et al., 2005; Moschetti et al., 2013; Moscetti et al., 2015; Tundoa et al., 2015). The TAXI–III inhibitory protein extracted and purified from transgenic durum wheat with *TaXI–III* gene could significantly reduce the cell death caused by xylanase FGSG_03624 and FGSG_10999 from *F. graminearum* in wheat lemma tissue and undifferentiated wheat cell suspension (Moscetti et al., 2015; Tundoa et al., 2015). Besides, compared with non-transgenic durum wheat, the mortality of lemma cells of transgenic durum wheat treated with xylanase FGSG_03624 and FGSG_10999 was significantly reduced (Moscetti et al., 2015; Tundoa et al., 2015). TAXI–III inhibitor still inhibited plant cell death induced by xylanase (FGSG_03624 and FGSG_10999) under thermal inactivation, which indicated that this function of the inhibitor was not affected by the structural changes of xylanase, including the destruction of active sites (Moscetti et al., 2015; Tundoa et al., 2015). Six XIs (TAXI–IA, TAXI–IAb, TAXI–IIA, TAXI–IIAb, TAXI–III, and TAXI–IV) and three *F. graminearum* xylanases (FGSG_03624, FGSG_10999, and FGSG_11487) were heterogeneously expressed in *P. pastoris*. The heterologously expressed proteins were extracted and injected into tobacco leaves. The results showed that six heterologous XIs could inhibit cell necrosis induced by xylanase FGSG_03624 and FGSG_10999, but could not inhibit cell necrosis induced by FGSG_11487. TAXI-type inhibitors can limit *F. graminis* infection by directly inhibiting xylanase activity and/or preventing xylanase from causing cell death. For these functions of inhibitors, compared with wild-type durum wheat, the FHB symptoms in transgenic durum wheat with *TaXI–III* gene within 3–11 days after inoculation with *F. graminae* were significantly reduced, and the fungal biomass in caryopsis of transgenic plants in the early and late stage of infection was slightly lower than that of wild-type plants (Moscetti et al., 2015).

## Conclusion

In this study, the wheat *TaXI* gene family was identified for the first time in the whole genome, and divided into six subfamilies. To better understand the structure and function of the *TaXI* genes, we analyzed their gene structure, protein motif and promoter *cis-*acting elements. Wheat polyploidization, tandem duplication and segmental duplication are the main reasons for the expansion of *TaXI* gene family members. The expression pattern of the *TaXI* gene in wheat has tissue specificity and diversity in response to stress. *TaXI* genes are involved in plant hormone-mediated metabolic processes and responses to abiotic and biotic stresses. We verified that TAXI–III inhibitors can directly inhibit xylanase activity and block xylanase-induced cell death, and also proved that other TAXI-type inhibitors of *XI-2* subfamily have similar functions. This study not only provides the scientific basis for a comprehensive understanding of wheat *TaXI* gene family, but also helps us to screen more candidate genes, and lays a foundation for further exploring the molecular mechanism of TAXI-type inhibitors inhibiting cell death caused by xylanase.

## Data Availability Statement

The datasets presented in this study can be found in online repositories. The names of the repository/repositories and accession number(s) can be found in the article/Supplementary Material.

## Author Contributions

YL designed the research, participated in all bioinformatic analysis, experiments, and analysis, and wrote the manuscript. NH and SW helped to sow wheat, plant tobacco, and inoculate wheat with *Fusarium graminis* and qRT-PCR. HS, CC, JL, and MR conceived and directed the study and helped to draft the manuscript. GS and CM revised and improved the draft. All authors contributed to the article and approved the submitted version.

## Conflict of Interest

The authors declare that the research was conducted in the absence of any commercial or financial relationships that could be construed as a potential conflict of interest.

## Publisher’s Note

All claims expressed in this article are solely those of the authors and do not necessarily represent those of their affiliated organizations, or those of the publisher, the editors and the reviewers. Any product that may be evaluated in this article, or claim that may be made by its manufacturer, is not guaranteed or endorsed by the publisher.

## Footnotes

1 https://wheat-urgi.versailles.inra.fr

2 http://pfam.xfam.org

3 https://www.ncbi.nlm.nih.gov/cdd/

4 https://www.expasy.org/structural_bioinformatics

5 http://www.cbs.dtu.dk/services/SignalP-4.0/

6 http://cello.life.nctu.edu.tw/cello.html

7 http://bioinformatics.psb.ugent.be/webtools/plantcare/html/

8 https://www.ncbi.nlm.nih.gov/

9 http://www.ebi.ac.uk

10 http://plants.ensembl.org/Triticum_aestivum/Info/Index

11 http://meme-suite.org/

12 http://www.wheat-expression.com/

## Supplementary Material

Supplementary Figure 1 Phylogenetic tree and *cis-*acting element analysis of the promoter regions of *TaXI* genes. **(A)** Phylogenetic relationships of *TaXI* genes. Red and yellow background colors represented homologous gene pairs or clusters, respectively. **(B)** *Cis-*acting element distribution in 2 kb promoter region upstream of CDS sequence of wheat *TaXI* gene family.

Supplementary Figure 2 Phylogenetic tree, gene structure, and architecture of conserved protein motifs in *TaXIs*. **(A)** Phylogenetic tree of 277 TAXI proteins. Colored boxes indicated the subfamily based on the phylogenetic analysis. **(B)** Exon/intron organization of *TaXI* genes. Exons, introns, and untranslated regions (UTRs) are indicated by yellow rectangles, gray lines, and green rectangles, respectively. **(C)** Distributions of conserved motifs in TAXI proteins. Twenty putative motifs were indicated in different colored boxes.

Supplementary Figure 3 DNS assay. Inhibition activities of six TAXIs crude protein extracted from *Pichia pastoris* culture medium against four *F. graminearum* xylanase activities [FGSG_03624 **(A,E,F,G)**, FGSG_10999 **(B,H,I,J)**, FGSG_11304 **(C,K,L,M)**, and FGSG_11487 **(D,N,O,P)**]. The color of the DNS solution changed (or did not change) with the increase of the amount of inhibitors.

Supplementary Table 1 The list of the nomenclature, characteristics of putative *TaXI* genes.

Supplementary Table 2 The Ka/Ks of segmentally and tandemly duplicated *TaXI* gene pairs.

Supplementary Table 3 The list of the putative motifs of TAXI protein.

Supplementary Table 4 Number of various *cis-*acting regulatory elements of *TaXI* genes in different subfamily.

Supplementary Table 5 Number of each *cis-*acting element in the promoter regions of *TaXI* genes.

Supplementary Table 6 The RNA-Seq atlas data of *TaXI* genes in different tissues at different developmental stages.

Supplementary Table 7 The RNA-Seq atlas data of *TaXI* genes under drought and heat stresses.

Supplementary Table 8 The RNA-Seq atlas data of *TaXI* genes under low-temperature treatment.

Supplementary Table 9 The RNA-Seq atlas data of *TaXI* genes under powdery mildew inoculation.

Supplementary Table 10 The RNA-Seq atlas data of *TaXI* genes under stripe rust inoculation.

Supplementary Table 11 The RNA-Seq atlas data of *TaXI* genes under *Fusarium graminearum* inoculation.

Supplementary Table 12 The list of the primers used in this study.

Supplementary Table 13 The absorbance value of the reaction solution of xylanase and inhibitor at A550 nm.
