# Supplementary figures and images for: Genome-Wide Identification of Triticum aestivum Xylanase Inhibitor Gene Family and Inhibitory Effects of XI-2 Subfamily Proteins on Fusarium graminearum GH11 Xylanase
Source: Front Plant Sci. 2021 Jul 26;12:665501. doi: 10.3389/fpls.2021.665501 (PMC8350787; doi:10.3389/fpls.2021.665501)

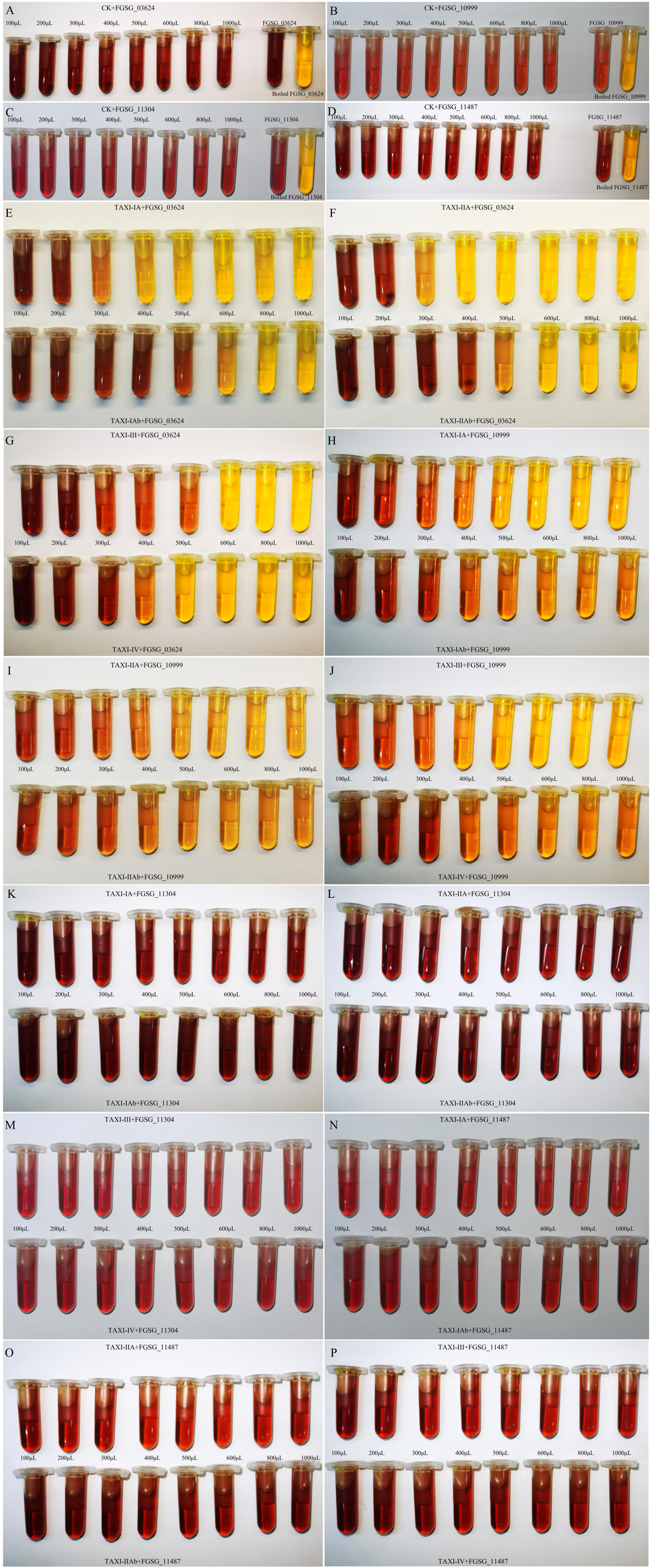

Supplement: Supplementary Figure 1 — Phylogenetic tree and cis-acting element analysis of the promoter regions of TaXI genes. (A) Phylogenetic relationships of TaXI genes. Red and yellow background colors represented homologous gene pairs or clusters, respectively. (B) Cis-acting element distribution in 2 kb promoter region upstream of CDS sequence of wheat TaXI gene family. [file Data_Sheet_1.zip › Supplementary Figures/Figure S3.jpg]
